# Potential urine proteomics biomarkers for primary nephrotic syndrome

**DOI:** 10.1186/s12014-017-9153-1

**Published:** 2017-05-16

**Authors:** Young Wook Choi, Yang Gyun Kim, Min-Young Song, Ju-Young Moon, Kyung-Hwan Jeong, Tae-Won Lee, Chun-Gyoo Ihm, Kang-Sik Park, Sang-Ho Lee

**Affiliations:** 1 Division of Nephrology, Department of Internal Medicine, Kyung Hee University School of Medicine, 892 Dongnam-ro, Gangdong-gu, Seoul, Korea; 2 Department of Physiology, Kyung Hee University School of Medicine, 26 Kyungheedae-ro, Dongdaemun-gu, Seoul, Korea

**Keywords:** Focal and segmental glomerulosclerosis, Minimal change disease, Membranous nephropathy, Nephrotic syndrome, Urine proteomics

## Abstract

**Background:**

Nephrotic syndrome (NS) is a nonspecific kidney disorder, commonly caused by minimal change disease (MCD), focal segmental glomerulosclerosis (FSGS), and membranous nephropathy (MN). Here we analyzed urinary protein profiles, aiming to discover disease-specific biomarkers of these three common diseases in NS.

**Methods:**

Sixteen urine samples were collected from patients with biopsy-proven NS and healthy controls. After removal of high-abundance proteins, the urinary protein profile was analyzed by LC–MS/MS to generate a discovery set. For validation, ELISA was used to analyze the selected proteins in 61 urine samples.

**Results:**

The discovery set included 228 urine proteins, of which 22 proteins were differently expressed in MCD, MN, and FSGS. Among these, C9, CD14, and SERPINA1 were validated by ELISA. All three proteins were elevated in MCD, MN, and FSGS groups compared with in IgA nephropathy and healthy controls. When a regression model was applied, receiver operating characteristic analysis clearly discriminated MCD from the other causative diseases in NS.

**Conclusions:**

We developed a disease-specific protein panel that discriminated between three main causes of NS. Through this pilot study, we suggest that urine proteomics could be a non-invasive and clinically available tool to discriminate MCD from MN and FSGS.

**Electronic supplementary material:**

The online version of this article (doi:10.1186/s12014-017-9153-1) contains supplementary material, which is available to authorized users.

## Background

Nephrotic syndrome (NS) is a nonspecific kidney disorder that can affect individuals of any age, and accounts for about 15% of cases of end-stage renal disease [1]. Membranous nephropathy (MN), minimal change disease (MCD), and focal and segmental glomerulosclerosis (FSGS) was three common diseases of NS in adults [2, 3]. The abnormality of visceral epithelial cell incurs these disorders but exact pathogenesis in each disease was different [4].

MCD and FSGS are sometimes considered part of the same disease spectrum based on their similar clinical features, pathological presentations, and genetic polymorphisms [5, 6]. However, patients with FSGS are more likely to be non-responders to glucocorticoid therapy and to progress to kidney failure [7, 8]. Moreover, FSGS characterized a variety of clinical courses and functional consequences with numerous monogenetic mutations, making it difficult to select therapy based on the current diagnostic tools and classifications [9–11]. While almost all patients with MCD sustain their renal function for their lifetime, two-thirds of patients with MN progress to end-stage renal disease within 5–15 years, and about half of patients with FSGS who do not respond to steroid treatment will eventually require renal replacement therapy [1, 12]. Idiopathic MN is the most common glomerulonephropathy in adults manifesting NS [2]; however, it is difficult to non-invasively diagnose MN and predict prognosis [13, 14]. Circulating phospholipase A2 receptor antibodies are associated with MN but not found in all patients with MN. Moreover, patients with MN show a variety of clinical courses and treatment responses [1, 15].

An accurate diagnosis is very important for predicting prognosis and planning treatment, and renal biopsy is a necessary test for definitive diagnosis. However, this is an invasive procedure that carries a risk of complications, such as hematoma, arteriovenous fistula, and infection. Moreover, renal biopsy is contraindicated in some clinical conditions [16]. Several biomarkers have been proposed to help with MCD and FSGS diagnosis, but none are currently clinically available [17, 18]. Thus, there remains a need for new biomarkers to enable definitive diagnosis with an easy and non-invasive technique.

As a specimen, urine has several advantages compared with serum or plasma. Urine can be collected easily and non-invasively, and is free from other components that can interfere with blood analysis, such as clotting factors, active enzymes, and immunoregulatory proteins. Therefore, urine proteomics have been developed as a valuable tool for biomarker discovery in various diseases [19–21]. Several studies already identified the utility of urinary proteome profiling to diagnose and follow cancer by using 2D difference in gel electrophoresis or LC–MS/MS [22, 23]. In renal diseases, on study showed the possibility of CD80 as a urinary biomarker to distinguish between patients with MCD and FSGS by western blotting and ELISA [17]. The expression of lysosome membrane protein-2 in urinary microvesicles was suggested to be a biomarker for the patients with idiopathic MN by LC–MS/MS [24]. However, proteomic approach for urine samples from the patients with massive proteinuria is still challenging because high abundant proteins from plasma interfere to discover the disease specific biomarkers.

To date, no reports describe biomarkers that can differentiate between common causative three diseases (MCD, MN, FSGS) in adult NS. In our present study, we used multiple affinity removal system (MARS) for removing 14 high abundance proteins and analyzed urine proteins with the aim of discovering disease-specific biomarkers of NS.

## Methods

### Patients and urine samples

Two sets of urines from different cohorts were collected for discovery and validation set. The discovery set composed of 16 urine samples from 16 patients with biopsy-proven 4 MCD, 4 MN, 4 FSGS, and 4 healthy controls. In the discovery set, each individual sample was used for the patients with NS but pooled sample was analyzed for healthy controls. Sixty one urine samples from 51 patients with biopsy-proven 13 MCD, 26 MN, 5 FSGS, 9 IgAN and 8 healthy controls were included in validation set (Table 1). All the glomerulopathy was not secondary types caused by drugs, infection and malignancies, but primary disease. All the patients and healthy controls were recruited from 2 hospitals (Kyung Hee University Hospital at Gangdong and Kyung Hee University Medical Center) in Seoul, Korea. The patients diagnosed diabetes, liver disease, infectious disease and lupus were excluded. The Institutional Review Board of Kyung Hee University Hospital at Gangdong and Kyung Hee University Medical Center acknowledged this study and informed consents were acquired from all patients and healthy controls. Clinical information was gathered at each center. Blood and spot urine samples were obtained at the morning first time on the day of the renal biopsy. Chronic kidney disease (CKD) was divided into five stage on estimated glomerular filtration rate (eGFR) according to Modification of Diet in Renal Disease (MDRD) formula (stage 1: >90 ml/min/1.73 m^2^, stage 2: 60–89 ml/min/1.73 m^2^, stage 3: 30–59 ml/min/1.73 m^2^, stage 4: 15–29 ml/min/1.73 m^2^, stage 5: <15 ml/min/1.73 m^2^) [25]. Urine preparation and procedure was performed as previously described [26]. Briefly, 50–100 mL of urines from participants were collected in sterile containers and centrifuged at 2000×*g* for 20 min under room temperature within 1 h after collection. The supernatant was isolated from the pellet. The pH of supernatant was adjusted to 7.0 with 1 M Tris–HCl (pH 7.0) and stored at −80 °C until analysis. Urinary protein and creatinine were quantified using Bio-Rad protein assay kit (Bio-Rad, USA) and Creatinine Parameter Assay Kit (R&D systems, MN, USA), respectively.

**Table 1:** Clinical characteristics in discovery and validation set

Group	N	Sex (M/F)	Age	24 h urine protein (g/L)	Serum creatinine (mg/dL)	CKD stage	Albumin (g/dL)	Cholesterol (mg/dL)
Discovery set (LC–MS/MS)								
HC	4	4/0	30 ± 2	N/A	N/A	N/A	N/A	N/A
MCD	4	1/3	49 ± 25	9.14 ± 3.6	1.2 ± 0.5	2.00 ± 0.31	2.1 ± 0.3	348 ± 88
MN	4	2/2	50 ± 16*	6.56 ± 5.6	0.8 ± 0.2	1.00 ± 0.00	3.9 ± 0.4^†^	172 ± 28^†^
FSGS	4	4/0	42 ± 23	6.17 ± 5.76	1.6 ± 0.4	2.67 ± 0.33	3.9 ± 0.4^†^	240 ± 75
Validation set (ELISA)								
HC	8	4/4	36 ± 13	N/A	N/A	N/A	N/A	N/A
IgAN	9	4/5	50 ± 10*	1.5 ± 1.1	0.98 ± 0.2	1.89 ± 0.20	4.0 ± 0.3^†^	217 ± 33^†^
MCD	13	5/8	49 ± 19	7.69 ± 3.2^‡^	1.0 ± 0.4	2.08 ± 0.18	2.5 ± 0.7	351 ± 81
MN	26	16/10	55 ± 13*	6.13 ± 3.1^‡^	0.98 ± 0.4	1.81 ± 0.15	3.2 ± 0.7^†,‡^	239 ± 78^†^
FSGS	5	1/4	27 ± 15^†, ‡, §^	6.26 ± 5.4^‡^	1.94 ± 0.5^†, ‡, §^	2.80 ± 0.20^‡, §^	3.8 ± 0.5^†^	249 ± 69^†^

CKD stage was estimated by MDRD equation

*N* patients number, *HC* healthy control, *MCD* minimal change disease, *MN* membranous nephropathy, *FSGS* focal segmental glomerulosclerosis, *IgAN* IgA nephropathy, *N/A* not applicable

* *p* < 0.05 versus HC; ^†^ *p* < 0.05 versus MCD; ^‡^ *p* < 0.05 versus IgAN; ^§^ *p* < 0.05 versus MN

### Urine preparation and procedure

Urine volume was adjusted by creatinine concentration of 30 mg/mL. Urinary proteins were concentrated by removing small molecular weight peptides and other materials (<10 kDa) after filtering the supernatant through Amicon Ultra centrifugal filtration tubes (Millipore, MA, USA), which were pre-equilibrated with 10 ml distil water and centrifuged at 3000×*g* for 10 min at 10 °C with swinging bucket rotors. Then, total 10 ml of urine supernatant with PBS was centrifuged for 60 min at 3000×*g* at 10 °C. The retentate was washed twice with 10 ml of 10 mM Tris–HCl (pH 7.0). The final volume of the retentate was formed to 400 μl with 10 mM Tris–HCl (pH 7.0) [26]. The sample were depleted of the 14 most abundant plasma proteins (albumin, IgG, antitrypsin, IgA, transferrin, haptoglobin, fibrinogen, alpha2-macroglobulin, alpha1-acid glycoprotein, IgM, apolipoprotein AI, apolipoprotein AII, complement C3, and transthyretin) with multiple affinity removal systems (Hu 14 MARS 4.6 × 100 mm, Agilent; Santa Clara, CA, USA) according to the manufacturer’s protocol [27] (Additional file 1: Figure S1). Depleted flow-through fractions were desalted using PD MiniTrap™ G-25 columns (GE Healthcare, UK) prior to the tryptic digestion following the manufacturer’s protocol.

### In-gel digestion and LC/MS/MS analysis

Rehydrated 50 µL of fractions after desalting of the low-abundance proteins were loaded. They were separated by SDS-PAGE on a 10% gel followed by staining with Coomassie blue. The bands were directly cut out of the gels, destained with 50% acetonitrile in 25 mM ammonium bicarbonate and dried in a speed vacuum concentrator. Dried gel pieces were reswollen with 25 mM ammonium bicarbonate (pH 8.0) containing 50 ng trypsin and incubated at 37 °C for 16-24 h. Supernatant peptide mixtures were extracted with 50% acetonitrile in 5% formic acid and dried in a speed vacuum concentrator. The tryptic-dried samples were analyzed using the Agilent HPLC-Chip/TOF MS system with the Agilent 1260 nano-LC system, HPLC Chip-cube MS interface and 6530 QTOF single quadrupole-TOF mass spectrometer (Agilent Technologies, Santa Clara, CA). The dried peptide samples were resuspended in 2% ACN/0.1% FA and concentrated on a Large-capacity HPLC Chip (Agilent Technologies). The HPLC chip incorporated an enrichment column (9 mm, 75 μm I.D., 160 nl) and a reverse-phase column (15 cm, 75 μm I.D., packed with Zorbax 300SB-C18 5 µm resins). The peptide separation was performed using a 110 min gradient of 3−45% buffer B (buffer A contained 0.1% FA, and buffer B contained 90% ACN/0.1% FA) at a flow rate of 300 nl/min. The MS and MS/MS data were acquired in the positive ion mode and data stored centroid mode. The chip spray voltage was set at 1850 V and carried with chip conditions. The drying gas temperature was set at 325 with a flow rate of 3.5 l/min. A medium isolation (4 m/z) window was used for precursor isolation. A collision energy with a slope of 3.7 V/100 Da and an offset of 2.5 V was used for fragmentation. Additionally, while the MS data were acquired over a mass range of 300–3000 m/z, the MS/MS data were acquired over a 50–2500 m/z mass range. Reference mass correction was performed using a reference mass of 922. Precursors were set in an exclusion list for 0.5 min after two MS/MS spectra. The MS/MS spectra were extracted using the Mass Hunter Qualitative Analysis B.05.00 software (Agilent Technologies, Santa Clara, CA) with default parameters, and the spectra were interpreted with Spectrum Mill MS proteomics workbench (Agilent Technologies, USA) by searching against the Uniprot/Swiss-Prot database (sp. Homo Sapiens, 07/24/2013). The raw MS/MS spectra were processed using the Spectrum Mill MS proteomics Workbench (Version A.03.03.084 SR4, Agilent Technologies). Data extraction, MS/MS search, and validation were processed with the following parameters. Extraction: Constant modification (carbamidomethylation of cysteine) and variable modification (oxidation of methionine); sequence tag length >1; mass range 300–4000 daltons; maximum charge +5; minimum signal-to-noise 25. MS/MS search: search mode (identity); Uniprot database (sp. Homo Sapiens, 7/24/2013); tryptic digestion; 2 maximum missed cleavages; minimum matched peak intensity 50%; precursor mass tolerance ± 20 ppm; product mass tolerance ±0.5 Da; ESI Q-TOF instrument. Result validation: protein validation results were done by filtering protein scores higher than 20; percentage of score peak intensity (SPI) higher than 70%. For label-free quantification analysis, we initially calculated the relative abundance using the MS total intensities of each peptide. The relative abundances of the proteins were calculated from the means of the relative MS total intensities of the corresponding unique peptides.

### ELISA validation

ELISA kit for complement 9 (C9) (Abcam, Cambridge, MA, USA); cluster of differentiation 44 (CD44) (Abcam, Cambridge, MA, USA); serpin peptidase inhibitor, clade A, member 1 (SERPINA1, alpha-1 antitrypsin) (Abcam, Cambridge, MA, USA); cluster of differentiation 14 (CD14) (Raybiotech, Norcross, GA, USA); Peptidoglycan recognition protein 2 (USCNK, Houston, TX, USA); heat shock 60 kda protein 1 (HSPD1) (proteintech, Chicago, IL, USA); SERPINA7 (BlueGene, Shanghai, China) were used and performed according to the manufacturers’ protocol. Urine samples were diluted 10–2000 fold in PBS buffer. All the plates were read by a VERSAmax microplate reader (Molecular Device, Sunnyvale, CA, USA). Urinary creatinine was quantified using Creatinine Parameter Assay Kit (R&D systems, MN, USA). The value of each ELISA was quantified after normalizing with creatinine (mg/ml).

### Statistical analysis

Differences between groups were determined using Kruskal–Wallis test. Then, we used non-parametric Mann–Whitney *U* test with *p* < 0.05 for the selection of the candidate biomarkers. The MS intensity data was transformed into log values. Receiver-operating characteristics (ROC) curves were constructed to assess the diagnostic values. All analyses were performed with SPSS statistical software (version 20; SPSS Inc., Chicago, IL, USA) and GraphPad Prism (version 5.0; GraphPad Software, San Diego California USA).

## Results

### Urinary protein identification with LC–MS/MS

**Fig. 1:**
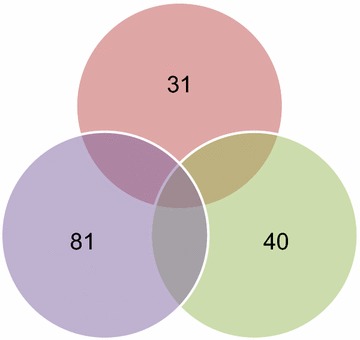
Venn diagram illustrated the numbers of proteins identified by LC–MS/MS in urine samples of patients with MCD, MN, and FSGS

We performed LC–MS/MS analysis of 16 urine samples from patients with NS (*n* = 4 each with MCD, MN, and FSGS) and healthy controls (*n* = 4). Table 1 shows the clinical characteristics. In discovery set, NS groups did not differ in age, sex, degree of proteinuria, or serum creatinine. Patients with MCD showed higher serum cholesterol and lower albumin. We identified a total of 228 urinary proteins, which are listed in Additional file 2. They included 21 proteins from healthy controls, 91 from MCD, 146 from MN, and 108 from FSGS (Fig. 1). Although the degree of proteinuria was similar, disease specific urinary proteins were existed as 31 in MCD, 81 in MN and 40 in FSGS.

### Differential expression of urine proteins in MCD, MN, and FSGS

**Fig. 2:**
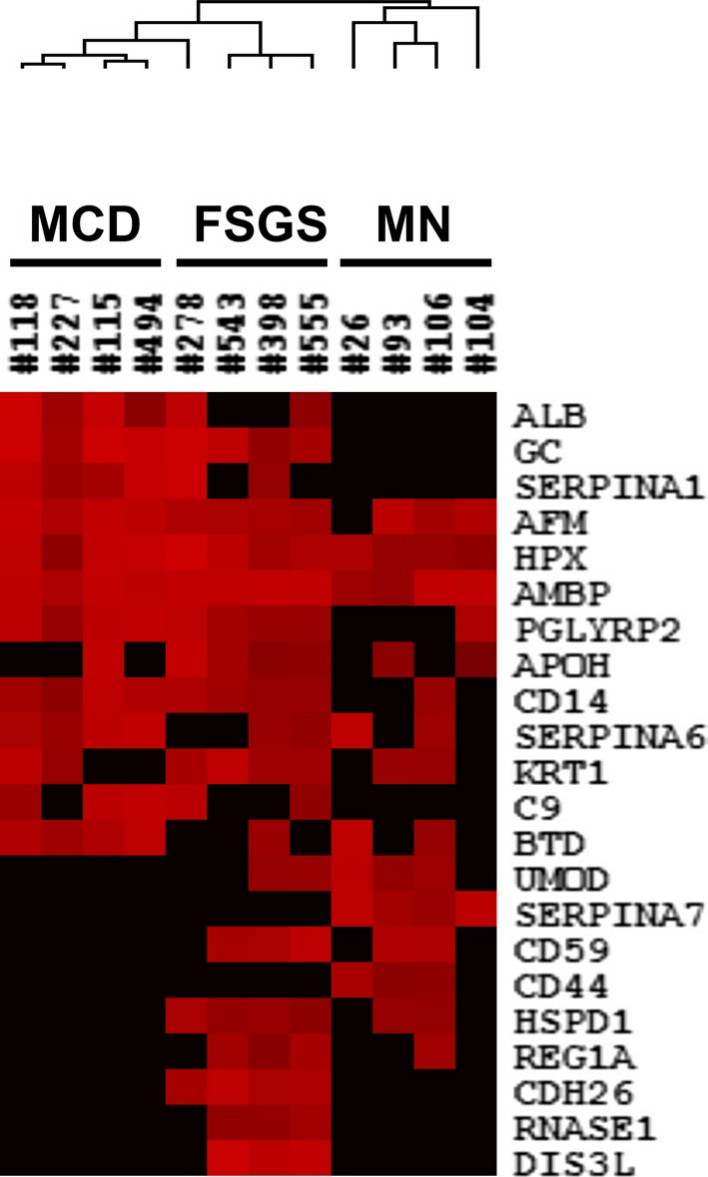
Hierarchical clustering of proteins identified in urine samples of patients with MCD, MN, and FSGS. The cluster reveals the differential expression of proteins among these diseases. The *box* denotes log_2_-transformed values of peaks intensity

To select candidate biomarkers from the 228 identified urine proteins, we used the Mann–Whitney *U* test. Twenty-two proteins were present in significantly different amounts among the three disease groups (MCD, MN, and FSGS) (Additional file 3). We performed hierarchical cluster analysis using log_2_-transformed values of peak intensity to analyze the expression patterns of these 22 proteins in the NS groups (Fig. 2). These results provided statistical evidence that CD14, C9, and SERPINA1 were specific to MCD; SERPINA7 and CD44 were specific to MN; and cadherin-like 26, ribonuclease, RNase A Family 1, and DIS3-like exonuclease 1 to FSGS.

### ELISA validation

We next performed ELISA assays for six proteins (C9, CD14, CD44, SERPINA1, and SERPINA 7, HSPD1) in an independent validation set including 8 healthy controls, 13 patients with MCD, 26 with MN, 5 with FSGS, and 9 with IgAN. In validation set, the patients with FSGS were younger and showed more deteriorated renal function (higher level of serum creatinine and CKD stage) than the patients with MCD and MN. The MCD group presented significantly low level of albumin and high level of cholesterole (Table 1). The NS patients manifested nephrotic range proteinuria, while the patients with IgAN showed non-nephrotic range proteinuria. The degree of proteinuria did not differ among the patients with MCD, MN, and FSGS.

**Fig. 3:**
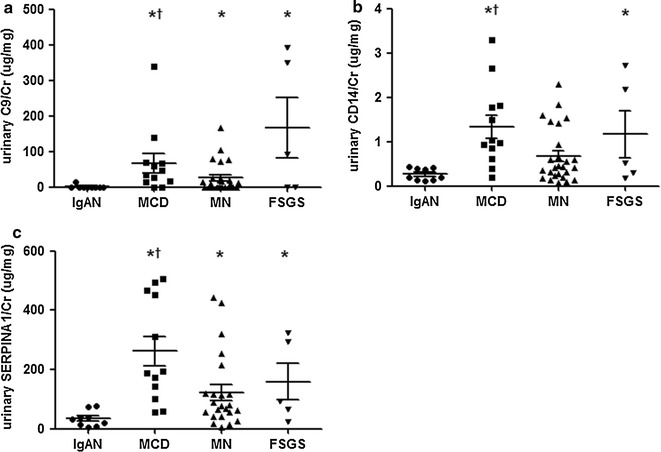
Validation of candidate biomarkers with ELISA. **a** C9, **b** CD14, and **c** SERPINA1 were measured by sandwich ELISA in a validation set. The ELISA levels were normalized to urine creatinine (µg/mg). *p < 0.05 versus IgAN, ^†^ *p* < 0.05 versus MN

Even though ELISA was performed for six disease-specific urine proteins, urinary levels of CD44, SERPINA7 and HSPD1 could not be detected with commercial ELISA Kits. Validated proteins (C9, CD14, SERPINA1) were almost nonexistent in the urine of healthy controls. Levels of C9 and SERPINA1 were significantly higher in the patients with NS than patients with IgAN. Only the level of CD14 was not significantly different in MN urine compared with IgAN urine (Fig. 3). The MCD group showed significantly higher mean urine concentrations of C9 (*p* = 0.048), CD14 (*p* = 0.014), and SERPINA1 (*p* = 0.009) than the MN group. FSGS could not be isolated from MCD and MN with individual protein of C9, CD14 and SERPINA1 (Fig. 3).

### Receiver operator characteristic (ROC) analysis

**Fig. 4:**
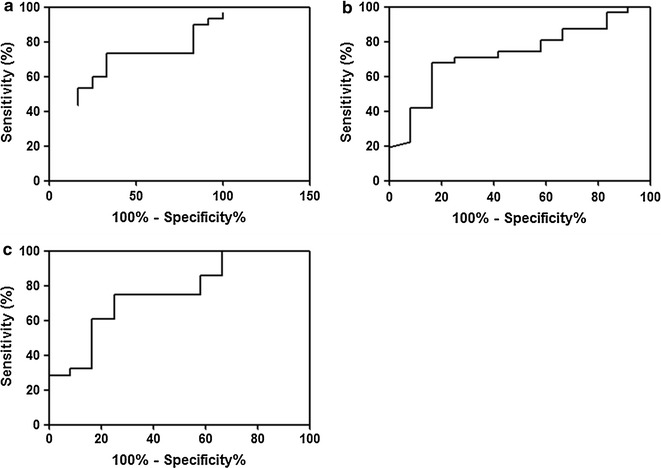
ROC curves with individual protein of **a** C9, **b** CD14 and **c** SERPINA1 between MCD and other NS diseases. The AUC of C9, CD14 and SERPINA1 was 0.650 (95% CI 0.473–0.827, *p* = 0.133), 0.719 (95% CI 0.559–0.879, *p* = 0.027) and 0.756 (95% CI 0.592–0.920, *p* = 0.011)

**Fig. 5:**
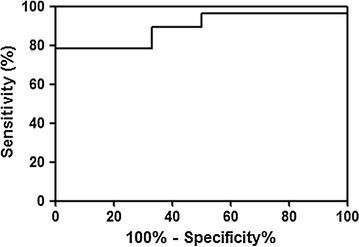
Logistic regression analysis with three proteins in discrimination of MCD from other NS diseases. The AUC of three proteins was 0.893 (95% CI 0.794–0.992, *p* < 0.001)

Receiver operator characteristic (ROC) analysis was performed to assess the use of C9, CD14 and SERPINA1 for differential diagnosis of MCD, because all of three proteins were specific for MCD (Additional file 3). Individual protein of CD14 and SERPINA1 could distinguish MCD from other NS diseases (MN and FSGS) (CD14: AUC 0.719, 95% CI 0.559–0.879, *p* = 0.027, SERPINA1: AUC 0.756, 95% CI 0.592–0.920, *p* = 0.011) (Fig. 4). Sum of three proteins showed highly predictive value for diagnosing MCD from other NS diseases (The area under curve (AUC) 0.893, 95% CI 0.794–0.992, *p* < 0.001) (Fig. 5). The logistic regression models with three proteins were applied to discriminate each disease from other disease among NS disease (Additional file 4: Figure S2). The AUC values significantly differed between MCD and MN (AUC 0.852, 95% CI 72.50–98.00, *p* = 0.001), MN and FSGS (AUC 0.817, 95% CI 0.599–1.000, *p* < 0.03), and MCD and FSGS (AUC 1, 95% CI 1.000–1.000, *p* < 0.001).

## Discussion

We aimed to identify biomarkers for individual NS groups using MS-based urine proteome analysis and further assessed whether these identified biomarkers retained statistical power following validation with independent samples. Our results demonstrated that urine proteome profiles can differentiate between three different types of NS and controls, and that the integrated three protein signatures—C9, CD14, and SERPINA1—can be used as clinical biomarkers for MCD.

Urine analysis has several advantages, including that urine samples are kidney-derived, and easily and non-invasively attainable. Several studies have used urine proteome profiling to find disease-specific biomarkers in glomerular diseases; however, no such biomarkers are clinically available yet [21, 28]. The urine proteome is more complex than the plasma proteome because tubular secretion and reabsorption could exist as well as glomerular filtration, all of which are involved in the determination of urine proteome. This pilot study might be the first trial to show the possibility that disease-specific urine proteins in three common diseases of NS could be existed. High-abundance proteins comprise over 90% of plasma proteins, and often obscure the detection of low-abundance proteins that might be useful as biomarkers. We used a MARS column to remove 14 high-abundance human plasma proteins from the urine samples prior to LC–MS/MS analysis. Although MARS column was applied, albumin was still detected in all urines of MCD group and several urines of FSGS group, but not detected in the urines of MN group. We thought the discrepancy was originated from the urinary characteristics in three diseases. Previous study revealed MCD urine was highest selective proteinuria for albumin, and was followed by other NS diseases [29]. Urine protein was larger in MCD group than other NS diseases though significant difference was not detected. Therefore, column could no thoroughly remove the relatively massive albumin, which was thought to be overflowed in the samples of MCD. In FSGS group, renal function was significantly worse than other NS diseases, besides sample size was small. Compared with idiopathic FSGS and MCD, secondary FSGS had small sized foot process width and various amount of proteinuria [30]. In this study, the value of validated urine protein was diverse in the FSGS group in which two patients revealed consistently high levels of three proteins, but other three were low levels of them. Edema as a main feature of NS was presented in two patients with elevated three proteins. Therefore, we thought three patients with relatively low amount of proteinuria could be secondary FSGS though we did not find identifiable secondary cause. In discovery set, albumin was detected in half of FSGS urine, thus the involvement of secondary FSGS could not be excluded also in discovery set.

This enabled the identification of 228 urine proteins from the patients with NS and normal controls, of which 22 proteins were identified as candidate biomarker showing differential expression in the three diseases. Among them, six proteins were selected for ELISA validation (CD14 for MCD and FSGS, SERPINA1 for MCD, C9 for MCD, CD44 for MN, SERPINA7 for MN, HSPD1 for FSGS). Unfortunately FSGS and MN specific proteins were not detected by commercially made ELISA kit and, MCD-specific urine proteins (C9, CD14, and SERPINA1) were validated in independent urine samples. These three proteins were substantially present in urine from patients with NS, but existed in very small amount in healthy controls and patients with IgAN. The patients with MN showed decreased levels of three proteins compared to the patients with MCD, which help to discriminate MN from MCD. No significant differences in three proteins were found for FSGS discrimination from other NS diseases. ROC curve analysis revealed that CD14 and SERPINA1 discriminated MCD from other NS diseases. Moreover, MCD was accurately distinguished from others with combined three proteins. When we tried to classify individual disease with combined three proteins, FSGS could not be differentiated from other two diseases. Nevertheless, ROC ROC curve showed clearly discrimination with combined three proteins, in which the confidence interval of the ROC was ranged from 1.00 to 1.00. ROC curve did not identify which disease characterized as high or low level of urinary protein, but only the level of combined proteins was different between MCD and FSGS. This error was given rise from the uncontrolled and small sized samples with variable clinical factors. However, three proteins were clearly distinguished MCD from other NS diseases by Mann–Whitney *U* test. Also, ROC curve clearly discriminated MCD from other NS (MN, FSGS) regardless of single or total application. Therefore, this study presented the possibility of diagnostic availability with urine proteomics in adult NS.

SERPINA1, also known as alpha-1 antitrypsin, has anti-proteolytic activity towards neutrophil elastase, proteinase 3, pancreatic elastase, trypsin, chymotrypsin, collagenases, and kallikrein [31, 32]. Increased serum levels of SERPINA1 are reported in various inflammatory diseases and acute kidney injury [33–35]. SERPINA1 is mainly derived from serum, and a small amount is formed from urine [36]. A recent study showed high amount of SERPINA1 in the urine of patients with glomerulonephritis could differentiate MCD and FSGS [37]. Similarly, our present data showed mean level of SERPINA1 is higher in the urine of patients with MCD compared to FSGS although significant difference was not found. Further investigations are needed to clarify the exact role of SERPIA1 in MCD.

C9 is an important protein in the formation of the membrane attacking complex C5b-9. Only scarce information is available regarding urine C9 in kidney diseases. One study demonstrated increased urinary C9 in patients with autosomal dominant polycystic kidney disease, with high expression in cyst-containing epithelial cells. The authors suggest that an over-activated alternative complement pathway might be related to disease progression [38]. In cases of proteinuria, it has been suggested that intratubular complement activation occurs via alternative pathways under increased ammonia production in proximal tubules [39]. We hypothesized that complement activation might be stimulated in the patients with severe proteinuria compared to patients with minimal proteinuria. Accordingly, urine C9 was elevated in the NS groups with nephrotic range proteinuria compared with in normal controls and in IgAN patients with non-nephrotic range proteinuria. Unlike CD14 and SERPINA1, the mean value of urine C9 was slightly elevated in the patients with FSGS relative to the patients with MCD. Among the three proteins, C9 has the largest molecular size: C9 (63.2 kDa) > SERPINA1 (46.7 kDa) > CD14 (40.1 kDa). A prior study showed that compared to MCD, FSGS was associated with more severe foot process effacement, which occurred to a varying degree of proteinuria and was dependent of the amount of proteinuria [30]. The proteinuria in MCD is highly selective albuminuria due to damaged charge barriers, while the proteinuria in FSGS has size selectivity due to increased pore size in basement membrane [40]. Therefore, it is likely that the relatively large C9 molecule was easily excreted in cases of FSGS through large pores and with severe foot process effacement. Only idiopathic NS was included in this experiment, but we could not rule out the possibility that secondary FSGS without identifiable cause could be included. Therefore, the large variability in C9 levels in FSGS may be related to diverse pore sizes in idiopathic and secondary FSGS.

CD14 is an innate immune system component that detects bacterial lipopolysaccharide as a co-receptor of Toll-like receptor 4 [41]. Elevated circulating CD14 is observed in patients with chronic kidney disease, and is reportedly associated with mortality among patients on dialysis [42]. Preterm infants showed increased urine CD14 [43], and high urine CD14 was a risk factor of renal fibrosis among patients with acute rejection after kidney transplantation [44]. Here we found significantly augmented urine CD14 levels in the patients with MCD relative to those with MN. Alterations of electronic change and hydrophobicity are critical for changing glomerular membrane characteristics [32]. Human CD14 has a hydrophobic cluster of several amino acids [45]. It has been suggested that glomerular-permeable CD14 may change the electronic charge of the glomerular membrane. However, the clinical meaning of urinary CD14 in NS remains unclear.

This study has several limitations. We did not presently assess other clinically important glomerular diseases that can lead to NS. MN and FSGS specific markers were not detected, thus we did not clearly confirm the differential diagnosis of MN and FSGS. This result cannot yet be generalized for widespread practice due to the small sized sample, especially in the FSGS group. To develop urine protein biomarkers as diagnostic tools, clinical usefulness have to be verified in a large sample sized study.

## Conclusion

This study demonstrated that a proteomic approach with urine samples could be a useful tool for develop biomarkers for NS. We carefully suggested that three candidate urine proteins—C9, CD14, and SERPINA1—could be the promising biomarkers for differentiating the patients with MCD from MN and FSGS.

## Additional files

**Additional file 1: Figure S1.** MARS chromatography of samples.

**Additional file 2: Table S1.** Total urine proteins in healthy controls and respective NS diseases.

**Additional file 3: Table S2.** Differently expressed 22 proteins in MCD, FSGS and MN.

**Additional file 4: Figure S2.** ROC curves after logistic analysis with combined three proteins in discrimination of three diseases of NS.

## Abbreviations

C9: complement 9
CD14: cluster of differentiation 14
FSGS: focal and segmental glomerulosclerosis
LC/MS/MS: liquid chromatography-tandem mass spectrometry
MCD: minimal change disease
MN: membranous nephropathy
NS: nephrotic syndrome
SERPINA1: serpin peptidase inhibitor, clade A, member 1

## References

[1] Maisonneuve P, Agodoa L, Gellert R, Stewart JH (2000). Distribution of primary renal diseases leading to end-stage renal failure in the United States, Europe, and Australia/New Zealand: results from an international comparative study. Am J Kidney Dis.

[2] Haas M, Meehan SM, Karrison TG, Spargo BH (1997). Changing etiologies of unexplained adult nephrotic syndrome: a comparison of renal biopsy findings from 1976–1979 and 1995–1997. Am J Kidney Dis.

[3] Gadegbeku CA, Gipson DS, Holzman LB, Ojo AO (2013). Design of the Nephrotic Syndrome Study Network (NEPTUNE) to evaluate primary glomerular nephropathy by a multidisciplinary approach. Kidney Int.

[4] Shankland SJ (2006). The podocyte’s response to injury: role in proteinuria and glomerulosclerosis. Kidney Int.

[5] Weber S, Gribouval O, Esquivel EL, Moriniere V (2004). NPHS2 mutation analysis shows genetic heterogeneity of steroid-resistant nephrotic syndrome and low post-transplant recurrence. Kidney Int.

[6] Vats A, Nayak A, Ellis D, Randhawa PS (2000). Familial nephrotic syndrome: clinical spectrum and linkage to chromosome 19q13. Kidney Int.

[7] Albaqumi M, Barisoni L (2008). Current views on collapsing glomerulopathy. J Am Soc Nephrol.

[8] Winn MP, Conlon PJ, Lynn KL, Howell DN (1999). Clinical and genetic heterogeneity in familial focal segmental glomerulosclerosis. International Collaborative Group for the Study of familial focal segmental glomerulosclerosis. Kidney Int.

[9] Barisoni L, Schnaper HW, Kopp JB (2007). A proposed taxonomy for the podocytopathies: a reassessment of the primary nephrotic diseases. Clin J Am Soc Nephrol.

[10] Hinkes B, Wiggins RC, Gbadegesin R, Vlangos CN (2006). Positional cloning uncovers mutations in PLCE1 responsible for a nephrotic syndrome variant that may be reversible. Nat Genet.

[11] Diomedi-Camassei F, Di Giandomenico S, Santorelli FM, Caridi G (2007). COQ2 nephropathy: a newly described inherited mitochondriopathy with primary renal involvement. J Am Soc Nephrol.

[12] Waldman M, Crew RJ, Valeri A, Busch J (2007). Adult minimal-change disease: clinical characteristics, treatment, and outcomes. Clin J Am Soc Nephrol.

[13] Beck LH, Bonegio RG, Lambeau G, Beck DM (2009). M-type phospholipase A2 receptor as target antigen in idiopathic membranous nephropathy. N Engl J Med.

[14] Kim YG, Choi YW, Kim SY, Moon JY (2015). Anti-phospholipase A2 receptor antibody as prognostic indicator in idiopathic membranous nephropathy. Am J Nephrol.

[15] Perna A, Schieppati A, Zamora J, Giuliano GA (2004). Immunosuppressive treatment for idiopathic membranous nephropathy: a systematic review. Am J Kidney Dis.

[16] Whittier WL, Korbet SM (2004). Renal biopsy: update. Curr Opin Nephrol Hypertens.

[17] Garin EH, Mu W, Arthur JM, Rivard CJ (2010). Urinary CD80 is elevated in minimal change disease but not in focal segmental glomerulosclerosis. Kidney Int.

[18] Meijers B, Maas RJ, Sprangers B, Claes K (2014). The soluble urokinase receptor is not a clinical marker for focal segmental glomerulosclerosis. Kidney Int.

[19] Kistler AD, Serra AL, Siwy J, Poster D (2013). Urinary proteomic biomarkers for diagnosis and risk stratification of autosomal dominant polycystic kidney disease: a multicentric study. PLoS ONE.

[20] Aregger F, Uehlinger DE, Witowski J, Brunisholz RA (2014). Identification of IGFBP-7 by urinary proteomics as a novel prognostic marker in early acute kidney injury. Kidney Int.

[21] Kalantari S, Rutishauser D, Samavat S, Nafar M (2013). Urinary prognostic biomarkers and classification of IgA nephropathy by high resolution mass spectrometry coupled with liquid chromatography. PLoS ONE.

[22] Weeks ME (2015). Urinary proteome profiling using 2D-DIGE and LC–MS/MS. Methods Mol Biol.

[23] Court M, Garin J, Masselon CD (2015). Urine sample preparation and fractionation for global proteome profiling by LC–MS. Methods Mol Biol.

[24] Rood IM, Merchant ML, Wilkey DW, Zhang T (2015). Increased expression of lysosome membrane protein 2 in glomeruli of patients with idiopathic membranous nephropathy. Proteomics.

[25] Levey AS, Coresh J, Greene T, Stevens LA (2006). Using standardized serum creatinine values in the modification of diet in renal disease study equation for estimating glomerular filtration rate. Ann Intern Med.

[26] Sigdel TK, Kaushal A, Gritsenko M, Norbeck AD (2010). Shotgun proteomics identifies proteins specific for acute renal transplant rejection. Proteom Clin Appl.

[27] He W, Huang C, Luo G, Dal Pra I (2012). A stable panel comprising 18 urinary proteins in the human healthy population. Proteomics.

[28] Thongboonkerd V (2008). Biomarker discovery in glomerular diseases using urinary proteomics. Proteom Clin Appl.

[29] Bazzi C, Petrini C, Rizza V, Arrigo G, D’Amico G (2000). A modern approach to selectivity of proteinuria and tubulointerstitial damage in nephrotic syndrome. Kidney Int.

[30] Deegens JK, Dijkman HB, Borm GF, Steenbergen EJ (2008). Podocyte foot process effacement as a diagnostic tool in focal segmental glomerulosclerosis. Kidney Int.

[31] Coakley RJ, Taggart C, O’Neill S, McElvaney NG (2001). Alpha1-antitrypsin deficiency: biological answers to clinical questions. Am J Med Sci.

[32] Lisowska-Myjak B (2005). AAT as a diagnostic tool. Clin Chim Acta.

[33] Ritchie RF, Palomaki GE, Neveux LM, Navolotskaia O (2000). Reference distributions for the positive acute phase proteins, alpha1-acid glycoprotein (orosomucoid), alpha1-antitrypsin, and haptoglobin: a comparison of a large cohort to the world’s literature. J Clin Lab Anal.

[34] Su L, Zhou R, Liu C, Wen B (2013). Urinary proteomics analysis for sepsis biomarkers with iTRAQ labeling and two-dimensional liquid chromatography-tandem mass spectrometry. J Trauma Acute Care Surg.

[35] Metzger J, Kirsch T, Schiffer E, Ulger P (2010). Urinary excretion of twenty peptides forms an early and accurate diagnostic pattern of acute kidney injury. Kidney Int.

[36] Candiano G, Musante L, Bruschi M, Petretto A (2006). Repetitive fragmentation products of albumin and alpha1-antitrypsin in glomerular diseases associated with nephrotic syndrome. J Am Soc Nephrol.

[37] Perez V, Ibernon M, Lopez D, Pastor MC (2014). Urinary peptide profiling to differentiate between minimal change disease and focal segmental glomerulosclerosis. PLoS ONE.

[38] Su Z, Wang X, Gao X, Liu Y (2014). Excessive activation of the alternative complement pathway in autosomal dominant polycystic kidney disease. J Intern Med.

[39] Matsuo S, Morita Y, Mizuno M, Nishikawa K, Yuzawa Y (1998). Proteinuria and damage to tubular cells—is complement a culprit?. Nephrol Dial Transplant.

[40] Guasch A, Deen WM, Myers BD (1993). Charge selectivity of the glomerular filtration barrier in healthy and nephrotic humans. J Clin Invest.

[41] Kitchens RL (2000). Role of CD14 in cellular recognition of bacterial lipopolysaccharides. Chem Immunol.

[42] Raj DS, Shah VO, Rambod M, Kovesdy CP, Kalantar-Zadeh K (2009). Association of soluble endotoxin receptor CD14 and mortality among patients undergoing hemodialysis. Am J Kidney Dis.

[43] Charlton JR, Norwood VF, Kiley SC, Gurka MJ, Chevalier RL (2012). Evolution of the urinary proteome during human renal development and maturation: variations with gestational and postnatal age. Pediatr Res.

[44] Matignon M, Ding R, Dadhania DM, Mueller FB (2014). Urinary cell mRNA profiles and differential diagnosis of acute kidney graft dysfunction. J Am Soc Nephrol.

[45] Debiec H, Guigonis V, Mougenot B, Decobert F (2002). Antenatal membranous glomerulonephritis due to anti-neutral endopeptidase antibodies. N Engl J Med.

