# Supplementary figures and images for: Potential urine proteomics biomarkers for primary nephrotic syndrome
Source: Clin Proteomics. 2017 May 16;14:18. doi: 10.1186/s12014-017-9153-1 (PMC5434615; doi:10.1186/s12014-017-9153-1)

## Slide 1
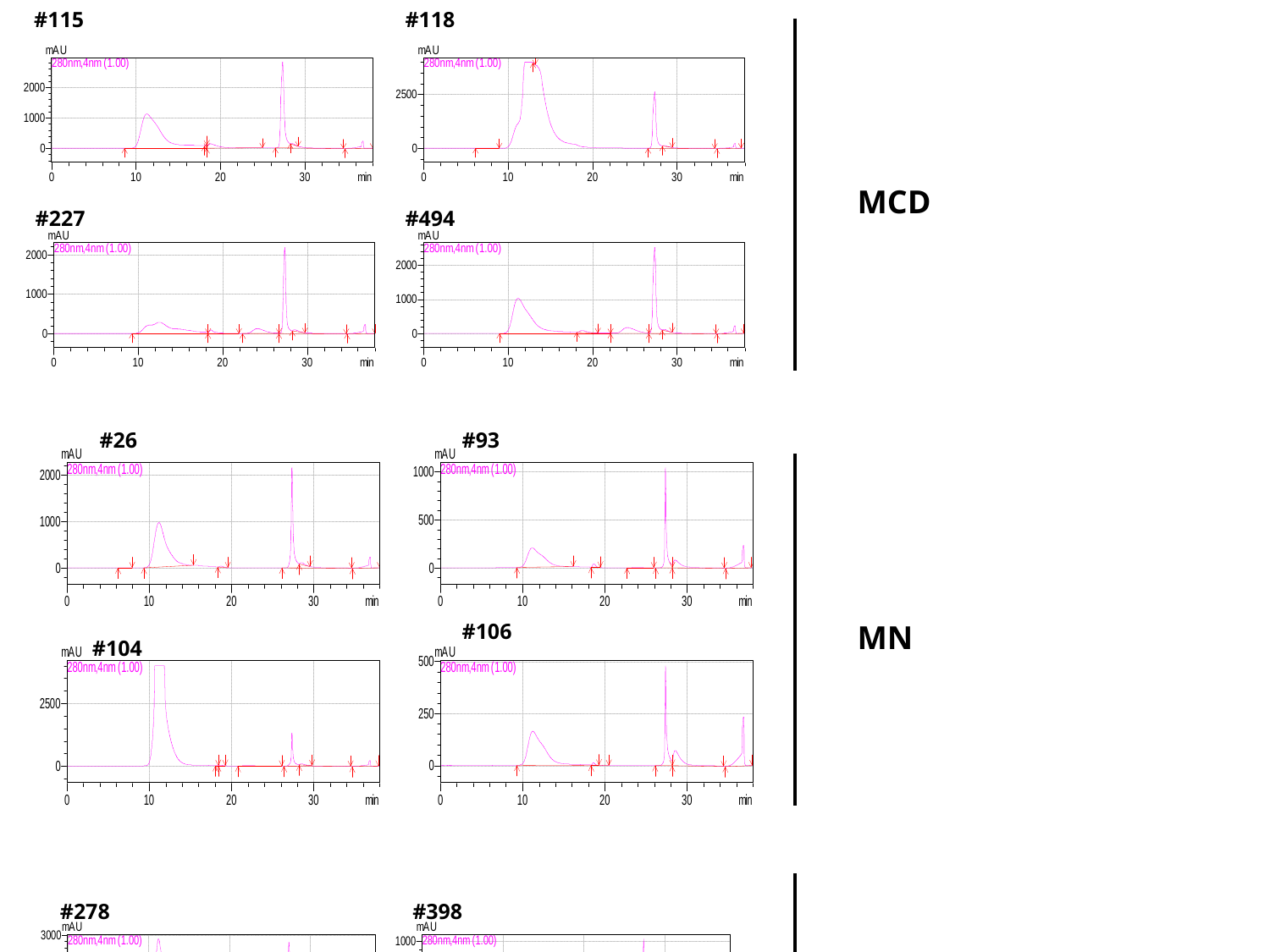

#115
#118
#227
#494
MCD
#26
#93
#106
#104
MN
#278
#398
#543
#555
FSGS

Supplement: Supplementary file 1 — Additional file 1: Figure S1. MARS chromatography of samples. [file 12014_2017_9153_MOESM1_ESM.pptx]

## Slide 1
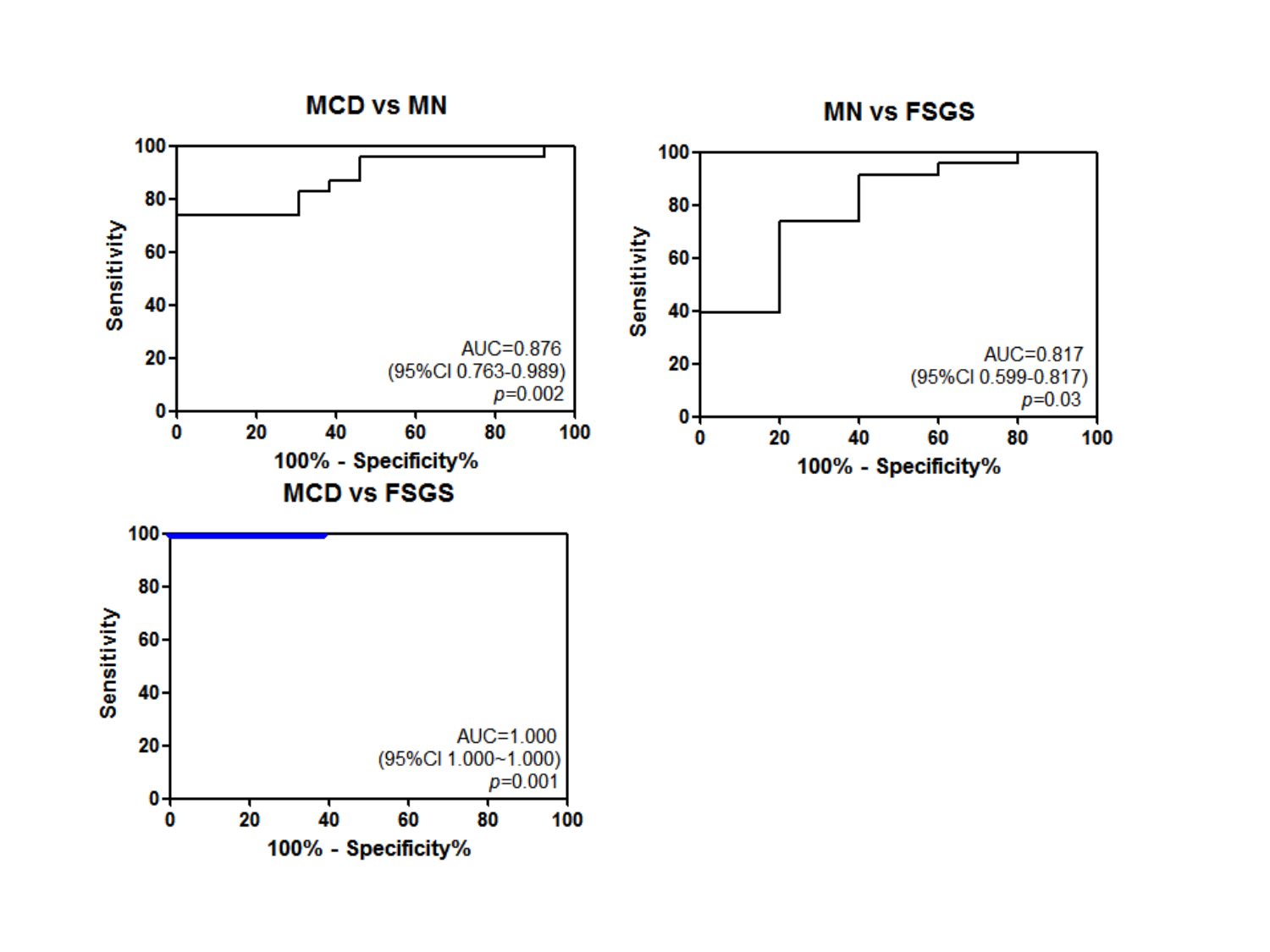

Supplement: Supplementary file 4 — Additional file 4: Figure S2. ROC curves after logistic analysis with combined three proteins in discrimination of three diseases of NS. [file 12014_2017_9153_MOESM4_ESM.pptx]
