# Supplementary material for: Potential urine proteomics biomarkers for primary nephrotic syndrome
Source: Clin Proteomics. 2017 May 16;14:18. doi: 10.1186/s12014-017-9153-1 (PMC5434615; doi:10.1186/s12014-017-9153-1)
Supplement: Supplementary file 2 — Additional file 2: Table S1. Total urine proteins in healthy controls and respective NS diseases. [file 12014_2017_9153_MOESM2_ESM.docx]

Additional file 2: Table 1. Total urine proteins

| **Accession No.** | **Gene  symbol** | **Protein Descriprion** | **kDa** | ***pI*** | **Healthy  control** | **MCD** | | | | **MGN** | | | | **FSGS** | | | |
| --- | --- | --- | --- | --- | --- | --- | --- | --- | --- | --- | --- | --- | --- | --- | --- | --- | --- |
|  |  |  |  |  |  | **115** | **118** | **227** | **494** | **104** | **106** | **26** | **93** | **278** | **398** | **543** | **555** |
| P02768 | ALB | Serum albumin | 69.4 | 5.9 | 17.6 | 22.1 | 23.3 | 17.8 | 15.8 | N/D | N/D | N/D | N/D | 20.3 | N/D | N/D | 16.0 |
| P0C0L5 | C4B | Complement C4-B | 192.8 | 6.7 | N/D | 21.3 | 18.7 | N/D | 19.5 | 18.4 | N/D | 18.6 | N/D | 21.2 | 17.6 | 17.2 | 17.3 |
| P02774 | GC | Vitamin D-binding protein | 53.0 | 5.4 | N/D | 23.0 | 22.9 | 18.4 | 22.3 | N/D | N/D | N/D | N/D | 22.9 | 16.4 | 20.5 | 18.8 |
| P00450 | CP | Ceruloplasmin | 122.2 | 5.4 | 16.3 | 21.6 | 20.8 | 18.0 | 21.9 | 21.0 | 17.7 | 21.5 | 18.7 | 21.3 | 17.7 | 19.7 | 17.6 |
| P01009 | SERPINA1 | Alpha-1-antitrypsin | 46.7 | 5.4 | N/D | 18.6 | 21.1 | 17.4 | 22.0 | N/D | N/D | N/D | N/D | 22.6 | 16.8 | N/D | N/D |
| P43652 | AFM | Afamin | 69.1 | 5.6 | 16.5 | 21.7 | 20.8 | 19.7 | 21.1 | 19.9 | 18.3 | N/D | 20.0 | 19.8 | 18.9 | 19.1 | 17.9 |
| P01011 | SERPINA3 | Alpha-1-antichymotrypsin | 47.7 | 5.3 | N/D | 19.9 | 21.2 | 20.0 | 20.1 | 20.8 | 18.9 | 19.0 | 19.1 | 21.1 | 19.1 | 20.6 | 19.0 |
| P01008 | SERPINC1 | Antithrombin-III | 52.6 | 6.3 | N/D | 21.3 | 18.2 | 15.9 | 21.5 | N/D | 14.4 | 20.0 | N/D | 22.0 | 16.6 | 17.4 | 17.2 |
| P25311 | AZGP1 | Zinc-alpha-2-glycoprotein | 34.3 | 5.7 | N/D | 21.7 | 21.1 | 20.6 | 21.2 | 21.5 | 20.0 | N/D | 19.9 | 21.0 | 20.2 | 20.7 | 19.7 |
| P04217 | A1BG | Alpha-1B-glycoprotein | 54.3 | 5.6 | 17.0 | 22.7 | 22.4 | 21.1 | 22.9 | 21.5 | 20.2 | 21.5 | 19.6 | 22.8 | 20.1 | 19.7 | 19.3 |
| P00734 | F2 | Prothrombin | 70.0 | 5.6 | N/D | 21.5 | 20.2 | N/D | 21.6 | N/D | N/D | 21.1 | N/D | 20.5 | N/D | 17.9 | 16.9 |
| P02790 | HPX | Hemopexin | 51.7 | 6.6 | 15.5 | 22.0 | 20.4 | 16.0 | 22.1 | 16.2 | 16.9 | 19.4 | 16.7 | 23.2 | 17.9 | 20.1 | 19.5 |
| P01024 | C3 | Complement C3 | 187.1 | 6.0 | N/D | 21.7 | N/D | N/D | 20.0 | N/D | N/D | 18.7 | N/D | 21.7 | 15.4 | 18.3 | 17.3 |
| P01042 | KNG1 | Kininogen-1 | 72.0 | 6.3 | N/D | 22.1 | 14.3 | 17.9 | 19.9 | 13.1 | 18.2 | N/D | N/D | 21.1 | 17.9 | 19.1 | 18.0 |
| P02760 | AMBP | Protein AMBP | 39.0 | 6.0 | 18.3 | 21.7 | 20.2 | 19.5 | 21.1 | 20.5 | 20.8 | 17.7 | 16.8 | 21.6 | 20.8 | 21.6 | 21.7 |
| P02787 | TF | Serotransferrin | 77.1 | 6.8 | 18.6 | N/D | 20.2 | N/D | 20.1 | N/D | N/D | N/D | N/D | N/D | N/D | N/D | N/D |
| Q96PD5 | PGLYRP2 | N-acetylmuramoyl-L-alanine amidase | 62.2 | 7.3 | N/D | 21.2 | 20.1 | 16.9 | 20.6 | 19.4 | N/D | N/D | N/D | 20.3 | 17.5 | 18.0 | 17.2 |
| Q14624 | ITIH4 | Inter-alpha-trypsin inhibitor heavy chain H4 | 103.4 | 6.5 | N/D | 21.0 | 20.7 | 16.8 | 20.1 | 16.4 | 17.3 | 18.0 | N/D | 20.8 | 17.6 | 18.9 | 17.6 |
| P01019 | AGT | Angiotensinogen | 53.2 | 5.9 | 14.9 | 22.0 | 20.9 | N/D | 21.1 | N/D | N/D | 14.7 | N/D | 21.4 | N/D | 18.4 | 17.2 |
| P02765 | AHSG | Alpha-2-HS-glycoprotein | 39.3 | 5.4 | N/D | 21.7 | 21.0 | 16.2 | 20.8 | 19.5 | 17.8 | N/D | 17.9 | 22.4 | 16.7 | 19.5 | 18.7 |
| P02749 | APOH | Beta-2-glycoprotein 1 | 38.3 | 8.3 | N/D | 21.6 | N/D | N/D | N/D | 13.5 | N/D | N/D | 15.6 | 20.9 | 15.6 | 17.9 | 16.2 |
| P02753 | RBP4 | Retinol-binding protein 4 | 23.0 | 5.8 | N/D | 20.4 | N/D | N/D | 20.5 | N/D | N/D | 22.7 | N/D | 21.6 | N/D | 18.9 | 19.5 |
| P02750 | LRG1 | Leucine-rich alpha-2-glycoprotein | 38.2 | 6.5 | 15.4 | 20.1 | 20.1 | 17.5 | 20.7 | 19.6 | 16.1 | 19.4 | 18.8 | 21.2 | 17.2 | 19.2 | 16.1 |
| P19823 | ITIH2 | Inter-alpha-trypsin inhibitor heavy chain H2 | 106.5 | 6.4 | N/D | 21.1 | 20.6 | N/D | 19.8 | N/D | N/D | N/D | 17.0 | 21.1 | N/D | N/D | 15.8 |
| P08571 | CD14 | Monocyte differentiation antigen CD14 | 40.1 | 5.8 | 15.4 | 20.2 | 18.2 | 16.3 | 19.9 | N/D | 17.0 | N/D | N/D | 19.6 | 16.8 | 17.8 | 16.9 |
| P08185 | SERPINA6 | Corticosteroid-binding globulin | 45.1 | 5.6 | N/D | 20.1 | 19.2 | 17.4 | 20.7 | N/D | 17.6 | 20.4 | N/D | N/D | 17.2 | N/D | 16.5 |
| P04264 | KRT1 | Keratin, type II cytoskeletal 1 | 66.0 | 8.2 | N/D | N/D | 21.0 | 16.8 | N/D | N/D | 16.9 | N/D | 17.2 | 18.8 | 17.8 | 21.5 | 17.8 |
| P02763 | ORM1 | Alpha-1-acid glycoprotein 1 | 23.5 | 4.9 | N/D | 20.6 | 21.4 | 16.9 | N/D | N/D | N/D | 16.3 | N/D | 22.7 | N/D | N/D | N/D |
| P36955 | SERPINF1 | Pigment epithelium-derived factor | 46.3 | 6.0 | N/D | 19.9 | N/D | N/D | 18.0 | N/D | N/D | N/D | N/D | 20.9 | N/D | 16.7 | N/D |
| P02748 | C9 | Complement component C9 | 63.2 | 5.4 | N/D | 21.0 | 17.4 | N/D | 20.7 | N/D | N/D | N/D | N/D | 21.2 | N/D | N/D | 16.3 |
| P00751 | CFB | Complement factor B | 85.5 | 6.7 | N/D | 20.6 | N/D | N/D | 16.5 | 15.5 | N/D | 20.3 | N/D | 20.7 | N/D | N/D | 17.1 |
| P05154 | SERPINA5 | Plasma serine protease inhibitor | 45.7 | 9.3 | N/D | 18.0 | N/D | 17.1 | 17.5 | 15.2 | 17.8 | N/D | 16.7 | 17.6 | N/D | N/D | N/D |
| P04004 | VTN | Vitronectin | 54.3 | 5.6 | N/D | 21.9 | N/D | N/D | 19.2 | N/D | N/D | N/D | N/D | 22.1 | N/D | 18.8 | N/D |
| P15586 | GNS | N-acetylglucosamine-6-sulfatase | 62.1 | 8.6 | N/D | N/D | N/D | 16.9 | N/D | N/D | 17.0 | N/D | N/D | N/D | 17.2 | 17.3 | N/D |
| P05156 | CFI | Complement factor I | 65.8 | 7.7 | N/D | 20.4 | N/D | N/D | N/D | N/D | N/D | N/D | N/D | 20.9 | N/D | N/D | N/D |
| P41222 | PTGDS | Prostaglandin-H2 D-isomerase | 21.0 | 7.7 | N/D | 22.1 | N/D | 20.0 | 21.3 | 20.6 | 20.4 | N/D | 19.1 | 19.8 | 20.3 | 21.1 | 20.6 |
| P51884 | LUM | Lumican | 38.4 | 6.2 | N/D | 20.2 | N/D | N/D | 20.2 | N/D | N/D | N/D | N/D | N/D | N/D | N/D | N/D |
| P43251 | BTD | Biotinidase | 61.1 | 5.8 | N/D | 19.6 | 19.8 | 17.8 | 20.3 | N/D | 16.8 | 20.2 | N/D | N/D | 17.4 | N/D | N/D |
| P01833 | PIGR | Polymeric immunoglobulin receptor | 83.3 | 5.6 | N/D | 20.2 | N/D | N/D | N/D | N/D | N/D | 19.4 | N/D | N/D | N/D | N/D | N/D |
| P04278 | SHBG | Sex hormone-binding globulin | 43.8 | 6.2 | N/D | 19.3 | N/D | N/D | 18.3 | N/D | N/D | N/D | N/D | N/D | N/D | N/D | N/D |
| P00746 | CFD | Complement factor D | 27.0 | 7.7 | N/D | 20.9 | N/D | N/D | N/D | N/D | N/D | N/D | N/D | N/D | N/D | N/D | N/D |
| P13645 | KRT10 | Keratin, type I cytoskeletal 10 | 58.8 | 5.1 | N/D | N/D | N/D | 16.4 | N/D | N/D | 16.0 | N/D | 17.2 | N/D | N/D | 20.9 | 17.1 |
| P17900 | GM2A | Ganglioside GM2 activator | 20.8 | 5.2 | N/D | N/D | N/D | 16.6 | N/D | N/D | 18.3 | N/D | 18.1 | N/D | 17.7 | 18.4 | 17.6 |
| P07339 | CTSD | Cathepsin D | 44.6 | 6.1 | N/D | 19.6 | N/D | 16.7 | N/D | N/D | N/D | N/D | N/D | N/D | N/D | N/D | N/D |
| Q06033 | ITIH3 | Inter-alpha-trypsin inhibitor heavy chain H3 | 99.8 | 5.5 | N/D | 19.5 | N/D | 16.1 | N/D | N/D | N/D | 19.4 | N/D | N/D | N/D | N/D | N/D |
| Q8WZ42 | TTN | Titin | 3816.0 | 6.0 | N/D | N/D | N/D | 17.2 | 19.7 | N/D | N/D | N/D | N/D | N/D | N/D | 19.4 | 15.5 |
| P35527 | KRT9 | Keratin, type I cytoskeletal 9 | 62.1 | 5.1 | N/D | N/D | N/D | 15.9 | N/D | N/D | 16.4 | N/D | 17.4 | N/D | 16.7 | 23.4 | 16.9 |
| P54108 | CRISP3 | Cysteine-rich secretory protein 3 | 27.6 | 8.1 | N/D | N/D | N/D | 16.9 | N/D | N/D | N/D | N/D | N/D | N/D | N/D | N/D | N/D |
| P06870 | KLK1 | Kallikrein-1 | 28.9 | 4.7 | N/D | N/D | N/D | 17.4 | N/D | N/D | 19.6 | N/D | 18.0 | N/D | 17.0 | N/D | N/D |
| Q9Y6V0 | PCLO | Protein piccolo | 553.3 | 6.1 | N/D | N/D | N/D | 14.9 | 19.3 | N/D | N/D | N/D | N/D | N/D | N/D | N/D | N/D |
| P08697 | SERPINF2 | Alpha-2-antiplasmin | 54.6 | 5.9 | N/D | 17.4 | N/D | N/D | N/D | N/D | N/D | N/D | N/D | 20.3 | N/D | N/D | N/D |
| Q04756 | HGFAC | Hepatocyte growth factor activator | 70.7 | 7.0 | N/D | N/D | N/D | N/D | 18.0 | N/D | N/D | N/D | N/D | N/D | N/D | N/D | N/D |
| P00441 | SOD1 | Superoxide dismutase [Cu-Zn] | 15.9 | 5.7 | N/D | N/D | N/D | 17.3 | N/D | N/D | 16.8 | N/D | N/D | N/D | 17.4 | N/D | 16.0 |
| Q16270 | IGFBP7 | Insulin-like growth factor-binding protein 7 | 29.1 | 8.3 | N/D | N/D | N/D | 16.8 | N/D | 12.7 | 17.2 | N/D | N/D | N/D | N/D | N/D | N/D |
| P98160 | HSPG2 | Basement membrane-specific heparan sulfate proteoglycan core protein | 468.8 | 6.1 | 15.5 | 17.3 | N/D | N/D | N/D | N/D | 17.2 | N/D | N/D | N/D | N/D | 19.2 | 18.4 |
| P82650 | MRPS22 | 28S ribosomal protein S22, mitochondrial | 41.3 | 7.7 | N/D | N/D | N/D | 16.5 | N/D | N/D | 16.8 | N/D | N/D | N/D | 16.4 | 16.9 | N/D |
| O15397 | IPO8 | Importin-8 | 119.9 | 5.0 | N/D | N/D | 21.2 | N/D | N/D | N/D | N/D | N/D | N/D | N/D | N/D | N/D | N/D |
| P19827 | ITIH1 | Inter-alpha-trypsin inhibitor heavy chain H1 | 101.4 | 6.3 | N/D | 19.9 | N/D | N/D | N/D | N/D | N/D | N/D | N/D | 18.3 | N/D | N/D | N/D |
| P07148 | FABP1 | Fatty acid-binding protein, liver | 14.2 | 6.6 | N/D | N/D | N/D | N/D | 16.8 | N/D | N/D | N/D | N/D | N/D | N/D | N/D | N/D |
| Q9BY12 | SCAPER | S phase cyclin A-associated protein in the endoplasmic reticulum | 158.2 | 7.2 | N/D | N/D | N/D | N/D | 20.2 | 20.1 | N/D | N/D | N/D | N/D | N/D | N/D | N/D |
| Q53HL2 | CDCA8 | Borealin | 31.3 | 9.9 | N/D | N/D | N/D | 18.9 | N/D | N/D | 18.6 | N/D | N/D | N/D | 19.5 | N/D | 19.1 |
| O43151 | TET3 | Methylcytosine dioxygenase TET3 | 179.4 | 7.0 | N/D | N/D | 18.4 | N/D | N/D | N/D | N/D | N/D | N/D | N/D | N/D | N/D | N/D |
| Q99675 | CGRRF1 | Cell growth regulator with RING finger domain protein 1 | 38.2 | 5.1 | N/D | N/D | 22.7 | N/D | N/D | N/D | N/D | N/D | N/D | N/D | N/D | N/D | N/D |
| Q7Z602 | GPR141 | Probable G-protein coupled receptor 141 | 35.5 | 9.4 | N/D | N/D | 21.9 | N/D | N/D | N/D | N/D | N/D | N/D | N/D | N/D | N/D | N/D |
| Q6ZN08 | ZNF66P | Putative zinc finger protein 66 | 51.4 | 9.4 | N/D | N/D | 18.7 | N/D | N/D | N/D | N/D | N/D | N/D | N/D | N/D | N/D | N/D |
| P0C604 | OR4A8P | Putative olfactory receptor 4A8 | 35.6 | 7.9 | N/D | 21.4 | N/D | N/D | N/D | N/D | N/D | N/D | N/D | N/D | N/D | N/D | N/D |
| Q6ZSZ5 | ARHGEF18 | Rho guanine nucleotide exchange factor 18 | 130.8 | 6.7 | N/D | N/D | N/D | N/D | 15.7 | N/D | N/D | N/D | N/D | N/D | N/D | N/D | N/D |
| P56715 | RP1 | Oxygen-regulated protein 1 | 240.7 | 5.6 | N/D | N/D | 21.3 | N/D | N/D | N/D | N/D | N/D | N/D | N/D | N/D | N/D | N/D |
| Q70EL1 | USP54 | Inactive ubiquitin carboxyl-terminal hydrolase 54 | 187.4 | 6.9 | N/D | N/D | 21.9 | N/D | N/D | N/D | N/D | N/D | N/D | N/D | N/D | N/D | N/D |
| P20929 | NEB | Nebulin | 772.9 | 9.1 | N/D | N/D | N/D | N/D | 13.3 | N/D | N/D | N/D | N/D | N/D | N/D | N/D | N/D |
| P12109 | COL6A1 | Collagen alpha-1(VI) chain | 108.5 | 5.3 | N/D | N/D | N/D | 15.1 | N/D | N/D | 16.2 | N/D | 16.7 | N/D | N/D | N/D | N/D |
| Q15477 | SKIV2L | Helicase SKI2W | 137.8 | 5.7 | N/D | N/D | 23.2 | N/D | N/D | N/D | N/D | N/D | N/D | N/D | N/D | N/D | N/D |
| P05546 | SERPIND1 | Heparin cofactor 2 | 57.1 | 6.4 | N/D | 19.9 | N/D | N/D | N/D | N/D | N/D | 19.3 | N/D | 18.7 | N/D | N/D | N/D |
| P52333 | JAK3 | Tyrosine-protein kinase JAK3 | 125.1 | 6.8 | N/D | N/D | N/D | N/D | 22.8 | N/D | N/D | N/D | N/D | N/D | N/D | N/D | N/D |
| Q13061 | TRDN | Triadin | 81.6 | 9.4 | N/D | 21.7 | N/D | N/D | N/D | N/D | N/D | N/D | N/D | N/D | N/D | N/D | 18.0 |
| Q9NQ79 | CRTAC1 | Cartilage acidic protein 1 | 71.4 | 5.0 | N/D | 15.9 | N/D | N/D | N/D | N/D | N/D | 14.3 | N/D | N/D | N/D | N/D | N/D |
| O14513 | NCKAP5 | Nck-associated protein 5 | 208.5 | 8.3 | N/D | N/D | N/D | N/D | 18.0 | N/D | N/D | N/D | N/D | N/D | N/D | N/D | N/D |
| P01034 | CST3 | Cystatin-C | 15.8 | 9.0 | N/D | N/D | N/D | 16.6 | N/D | N/D | 16.9 | N/D | 17.1 | 20.8 | 16.7 | N/D | N/D |
| P53814 | SMTN | Smoothelin | 99.1 | 9.2 | N/D | N/D | N/D | N/D | 15.3 | N/D | N/D | N/D | N/D | N/D | N/D | N/D | N/D |
| O75116 | ROCK2 | Rho-associated protein kinase 2 | 160.9 | 5.8 | N/D | 18.5 | N/D | N/D | N/D | N/D | N/D | N/D | N/D | N/D | N/D | N/D | N/D |
| Q8IXK0 | PHC2 | Polyhomeotic-like protein 2 | 90.7 | 8.9 | N/D | N/D | N/D | N/D | 22.8 | N/D | N/D | N/D | N/D | N/D | N/D | N/D | N/D |
| P78527 | PRKDC | DNA-dependent protein kinase catalytic subunit | 469.1 | 6.8 | N/D | N/D | N/D | N/D | 24.4 | N/D | N/D | N/D | N/D | N/D | N/D | N/D | N/D |
| Q5TCQ9 | MAGI3 | Membrane-associated guanylate kinase,  WW and PDZ domain-containing protein 3 | 165.6 | 8.2 | N/D | 24.8 | N/D | N/D | N/D | N/D | N/D | N/D | N/D | N/D | N/D | N/D | N/D |
| P49792 | RANBP2 | E3 SUMO-protein ligase RanBP2 | 358.2 | 5.9 | N/D | N/D | N/D | 15.0 | N/D | N/D | N/D | N/D | N/D | N/D | N/D | N/D | N/D |
| Q9NSY1 | BMP2K | BMP-2-inducible protein kinase | 129.2 | 6.1 | N/D | N/D | 16.2 | N/D | N/D | N/D | N/D | N/D | N/D | N/D | N/D | N/D | N/D |
| P27487 | DPP4 | Dipeptidyl peptidase 4 | 88.3 | 5.7 | N/D | N/D | N/D | 16.4 | N/D | N/D | 16.4 | N/D | N/D | N/D | 16.4 | N/D | N/D |
| Q01459 | CTBS | Di-N-acetylchitobiase | 43.8 | 6.2 | N/D | N/D | N/D | 16.4 | N/D | N/D | 16.1 | N/D | 16.1 | N/D | N/D | N/D | N/D |
| P36776 | LONP1 | Lon protease homolog, mitochondrial | 106.5 | 6.0 | N/D | N/D | N/D | N/D | 22.0 | N/D | N/D | N/D | N/D | N/D | N/D | N/D | N/D |
| Q9UNN8 | PROCR | Endothelial protein C receptor | 26.7 | 6.7 | N/D | N/D | N/D | 15.0 | N/D | N/D | 16.0 | N/D | 15.8 | N/D | N/D | N/D | N/D |
| O95271 | TNKS | Tankyrase-1 | 142.0 | 6.6 | N/D | N/D | 21.8 | N/D | N/D | N/D | N/D | N/D | N/D | N/D | N/D | N/D | N/D |
| P18428 | LBP | Lipopolysaccharide-binding protein | 53.4 | 6.2 | N/D | 17.8 | N/D | N/D | N/D | N/D | N/D | 18.6 | N/D | 20.2 | N/D | N/D | N/D |
| P68871 | HBB | Hemoglobin subunit beta | 16.0 | 6.7 | N/D | N/D | N/D | N/D | N/D | 24.0 | 20.6 | N/D | N/D | N/D | N/D | N/D | N/D |
| P69905 | HBA1 | Hemoglobin subunit alpha | 15.3 | 8.7 | N/D | N/D | N/D | N/D | N/D | 23.6 | 19.5 | N/D | N/D | N/D | N/D | N/D | N/D |
| P02675 | FGB | Fibrinogen beta chain | 55.9 | 8.5 | N/D | N/D | N/D | N/D | N/D | 19.4 | N/D | N/D | N/D | N/D | N/D | N/D | N/D |
| P07911 | UMOD | Uromodulin | 69.8 | 5.1 | N/D | N/D | N/D | N/D | N/D | N/D | 17.6 | 20.1 | 16.6 | N/D | 16.9 | N/D | 17.1 |
| P00915 | CA1 | Carbonic anhydrase 1 | 28.9 | 6.6 | N/D | N/D | N/D | N/D | N/D | 20.8 | 15.8 | N/D | N/D | N/D | 17.3 | 17.3 | N/D |
| P02679 | FGG | Fibrinogen gamma chain | 51.5 | 5.4 | N/D | N/D | N/D | N/D | N/D | 19.5 | N/D | N/D | N/D | N/D | N/D | N/D | N/D |
| O00187 | MASP2 | Mannan-binding lectin serine protease 2 | 75.7 | 5.4 | N/D | N/D | N/D | N/D | N/D | 17.3 | 18.4 | N/D | N/D | N/D | N/D | N/D | N/D |
| P05543 | SERPINA7 | Thyroxine-binding globulin | 46.3 | 5.9 | N/D | N/D | N/D | N/D | N/D | 20.2 | 17.1 | 21.4 | 18.2 | N/D | N/D | N/D | N/D |
| P12830 | CDH1 | Cadherin-1 | 97.5 | 4.6 | N/D | N/D | N/D | N/D | N/D | N/D | 16.8 | N/D | N/D | N/D | N/D | N/D | 16.7 |
| P54802 | NAGLU | Alpha-N-acetylglucosaminidase | 82.3 | 6.2 | N/D | N/D | N/D | N/D | N/D | N/D | 16.3 | N/D | 16.3 | N/D | N/D | N/D | N/D |
| P13987 | CD59 | CD59 glycoprotein | 14.2 | 6.0 | N/D | N/D | N/D | N/D | N/D | N/D | 19.6 | N/D | 19.9 | N/D | 19.0 | 18.9 | 20.3 |
| Q9NZP8 | C1RL | Complement C1r subcomponent-like protein | 53.5 | 6.8 | N/D | N/D | N/D | N/D | N/D | N/D | 16.3 | N/D | 17.2 | N/D | 16.6 | N/D | N/D |
| P04745 | AMY1A | Alpha-amylase 1 | 57.8 | 6.5 | N/D | N/D | N/D | N/D | N/D | N/D | 16.4 | N/D | 15.6 | N/D | N/D | N/D | N/D |
| P32119 | PRDX2 | Peroxiredoxin-2 | 21.9 | 5.7 | N/D | N/D | N/D | N/D | N/D | 20.6 | N/D | N/D | N/D | N/D | N/D | N/D | N/D |
| P02774-3 | GC | Isoform 3 of Vitamin D-binding protein | 55.1 | 5.6 | 16.4 | N/D | N/D | N/D | N/D | N/D | N/D | 17.7 | 17.0 | N/D | N/D | N/D | N/D |
| P61769 | B2M | Beta-2-microglobulin | 13.7 | 6.1 | N/D | N/D | N/D | N/D | N/D | N/D | 17.2 | 15.8 | N/D | N/D | N/D | N/D | N/D |
| O43451 | MGAM | Maltase-glucoamylase, intestinal | 209.9 | 5.3 | N/D | N/D | N/D | N/D | N/D | N/D | 16.6 | N/D | N/D | N/D | N/D | N/D | N/D |
| P16070 | CD44 | CD44 antigen | 81.5 | 5.1 | N/D | N/D | N/D | N/D | N/D | N/D | 16.1 | 19.0 | 15.8 | N/D | N/D | N/D | N/D |
| Q12907 | LMAN2 | Vesicular integral-membrane protein VIP36 | 40.2 | 6.5 | 16.3 | N/D | N/D | N/D | N/D | N/D | 17.2 | N/D | 18.0 | N/D | N/D | N/D | N/D |
| Q9UBR2 | CTSZ | Cathepsin Z | 33.9 | 6.7 | N/D | N/D | N/D | N/D | N/D | N/D | 15.6 | N/D | N/D | N/D | N/D | N/D | N/D |
| P22352 | GPX3 | Glutathione peroxidase 3 | 25.4 | 8.3 | N/D | N/D | N/D | N/D | N/D | N/D | N/D | 20.0 | 15.3 | N/D | N/D | N/D | N/D |
| P06396 | GSN | Gelsolin | 85.7 | 5.9 | N/D | N/D | N/D | N/D | N/D | N/D | 16.4 | 16.7 | N/D | N/D | N/D | 18.6 | 17.8 |
| P01031 | C5 | Complement C5 | 188.3 | 6.1 | N/D | N/D | N/D | N/D | N/D | N/D | N/D | 18.6 | N/D | N/D | N/D | N/D | N/D |
| P10809 | HSPD1 | 60 kDa heat shock protein, mitochondrial | 61.1 | 5.7 | N/D | N/D | N/D | N/D | N/D | N/D | 16.7 | N/D | 16.8 | 19.0 | 17.1 | 16.7 | 15.8 |
| P10619 | CTSA | Lysosomal protective protein | 54.5 | 6.2 | N/D | N/D | N/D | N/D | N/D | N/D | N/D | N/D | 17.0 | N/D | N/D | N/D | N/D |
| P00918 | CA2 | Carbonic anhydrase 2 | 29.2 | 6.9 | N/D | N/D | N/D | N/D | N/D | 18.6 | N/D | N/D | N/D | N/D | N/D | N/D | N/D |
| E7ETH0 | CFI | Complement factor I light chain | 66.6 | 7.9 | N/D | N/D | N/D | N/D | N/D | N/D | N/D | 18.5 | N/D | N/D | N/D | N/D | N/D |
| P10643 | C7 | Complement component C7 | 93.5 | 6.1 | N/D | N/D | N/D | N/D | N/D | N/D | N/D | 19.4 | N/D | N/D | N/D | N/D | N/D |
| P15151 | PVR | Poliovirus receptor | 45.3 | 6.1 | N/D | N/D | N/D | N/D | N/D | N/D | N/D | N/D | 15.5 | N/D | N/D | N/D | N/D |
| P15309-2 | ACPP | Isoform 2 of Prostatic acid phosphatase | 48.3 | 6.5 | N/D | N/D | N/D | N/D | N/D | N/D | N/D | N/D | 17.2 | N/D | N/D | N/D | N/D |
| P02788 | LTF | Lactotransferrin | 78.2 | 8.5 | N/D | N/D | N/D | N/D | N/D | N/D | 15.9 | N/D | N/D | N/D | N/D | N/D | N/D |
| O75594 | PGLYRP1 | Peptidoglycan recognition protein 1 | 21.7 | 8.9 | 15.0 | N/D | N/D | N/D | N/D | N/D | 17.5 | N/D | 16.6 | N/D | 17.2 | N/D | 17.8 |
| P07357 | C8A | Complement component C8 alpha chain | 65.2 | 6.1 | N/D | N/D | N/D | N/D | N/D | N/D | N/D | 18.1 | N/D | N/D | N/D | N/D | N/D |
| Q96FE7 | PIK3IP1 | Phosphoinositide-3-kinase-interacting protein 1 | 28.2 | 4.9 | N/D | N/D | N/D | N/D | N/D | N/D | 17.0 | N/D | 17.1 | N/D | 16.9 | N/D | 17.1 |
| G3V357 | RNASE1 | Ribonuclease pancreatic | 13.0 | 8.1 | N/D | N/D | N/D | N/D | N/D | N/D | N/D | N/D | 16.2 | N/D | N/D | N/D | N/D |
| P60174 | TPI1 | Triosephosphate isomerase | 30.8 | 5.7 | N/D | N/D | N/D | N/D | N/D | N/D | N/D | N/D | 14.0 | N/D | N/D | N/D | N/D |
| J3KPA1 | CRISP3 | Cysteine-rich secretory protein 3 | 31.0 | 7.8 | N/D | N/D | N/D | N/D | N/D | N/D | N/D | N/D | 16.7 | N/D | N/D | N/D | N/D |
| P15169 | CPN1 | Carboxypeptidase N catalytic chain | 52.3 | 6.9 | N/D | N/D | N/D | N/D | N/D | N/D | N/D | N/D | 16.3 | N/D | N/D | N/D | N/D |
| E9PPQ4 | FTH1 | Ferritin heavy chain (Fragment) | 6.7 | 5.5 | N/D | N/D | N/D | N/D | N/D | N/D | N/D | N/D | 16.6 | N/D | N/D | N/D | N/D |
| P05451 | REG1A | Lithostathine-1-alpha | 18.7 | 5.7 | N/D | N/D | N/D | N/D | N/D | N/D | 17.9 | N/D | N/D | N/D | 15.4 | 18.2 | 18.6 |
| K7ERG9 | CFD | Complement factor D | 27.9 | 6.8 | N/D | N/D | N/D | N/D | N/D | N/D | N/D | 19.9 | N/D | N/D | N/D | N/D | N/D |
| O94919 | ENDOD1 | Endonuclease domain-containing 1 protein | 55.0 | 5.6 | N/D | N/D | N/D | N/D | N/D | N/D | 16.8 | N/D | N/D | N/D | 16.7 | N/D | N/D |
| P08174 | CD55 | Complement decay-accelerating factor | 41.4 | 7.8 | N/D | N/D | N/D | N/D | N/D | N/D | 17.4 | N/D | N/D | N/D | 16.3 | N/D | 17.8 |
| Q9Y3D8 | TAF9 | Adenylate kinase isoenzyme 6 | 20.1 | 4.5 | N/D | N/D | N/D | N/D | N/D | N/D | N/D | 24.2 | 22.2 | N/D | N/D | N/D | N/D |
| Q6UXB8 | PI16 | Peptidase inhibitor 16 | 49.5 | 5.2 | N/D | N/D | N/D | N/D | N/D | N/D | 16.6 | N/D | N/D | N/D | N/D | N/D | 16.1 |
| Q8TF72 | SHROOM3 | Protein Shroom3 | 216.9 | 7.9 | N/D | N/D | N/D | N/D | N/D | N/D | N/D | N/D | 14.3 | N/D | N/D | N/D | N/D |
| P29317 | EPHA2 | Ephrin type-A receptor 2 | 108.3 | 5.9 | N/D | N/D | N/D | N/D | N/D | 21.9 | N/D | N/D | N/D | N/D | N/D | N/D | N/D |
| Q53LP3 | SOWAHC | Ankyrin repeat domain-containing protein SOWAHC | 55.7 | 6.6 | N/D | N/D | N/D | N/D | N/D | N/D | N/D | N/D | 17.5 | N/D | N/D | N/D | N/D |
| E7ERU0 | DST | Dystonin | 615.7 | 5.5 | 18.8 | N/D | N/D | N/D | N/D | N/D | N/D | N/D | 18.2 | N/D | N/D | N/D | N/D |
| P08118 | MSMB | Beta-microseminoprotein | 12.9 | 5.4 | N/D | N/D | N/D | N/D | N/D | N/D | N/D | N/D | 16.0 | N/D | N/D | N/D | N/D |
| Q08380 | LGALS3BP | Galectin-3-binding protein | 65.3 | 5.1 | N/D | N/D | N/D | N/D | N/D | N/D | 14.0 | N/D | N/D | N/D | N/D | N/D | N/D |
| O75106 | AOC2 | Retina-specific copper amine oxidase | 83.7 | 6.5 | N/D | N/D | N/D | N/D | N/D | N/D | 15.6 | N/D | 15.3 | N/D | N/D | N/D | N/D |
| E9PK50 | ABCC8 | ATP-binding cassette sub-family C member 8 | 112.0 | 8.9 | N/D | N/D | N/D | N/D | N/D | N/D | N/D | 20.2 | N/D | N/D | N/D | N/D | N/D |
| Q9NTG1 | PKDREJ | Polycystic kidney disease and receptor for egg jelly-related protein | 255.5 | 9.3 | N/D | N/D | N/D | N/D | N/D | N/D | N/D | N/D | 24.0 | N/D | N/D | N/D | N/D |
| P02792 | FTL | Ferritin light chain | 20.0 | 5.5 | N/D | N/D | N/D | N/D | N/D | N/D | 18.6 | N/D | N/D | N/D | 18.7 | 17.2 | N/D |
| P55000 | SLURP1 | Secreted Ly-6/uPAR-related protein 1 | 11.2 | 5.2 | N/D | N/D | N/D | N/D | N/D | N/D | 15.5 | N/D | N/D | N/D | N/D | N/D | N/D |
| Q8WVN6 | SECTM1 | Secreted and transmembrane protein 1 | 27.0 | 7.0 | N/D | N/D | N/D | N/D | N/D | N/D | 17.3 | N/D | N/D | N/D | N/D | N/D | N/D |
| Q9NRC6 | SPTBN5 | Spectrin beta chain, non-erythrocytic 5 | 416.8 | 6.2 | N/D | N/D | N/D | N/D | N/D | N/D | N/D | N/D | 21.4 | N/D | N/D | N/D | N/D |
| Q96RT7 | TUBGCP6 | Gamma-tubulin complex component 6 | 200.5 | 5.9 | N/D | N/D | N/D | N/D | N/D | 20.4 | N/D | N/D | N/D | N/D | N/D | N/D | N/D |
| Q7RTY7 | OVCH1 | Ovochymase-1 | 125.1 | 8.7 | N/D | N/D | N/D | N/D | N/D | N/D | N/D | 17.5 | N/D | N/D | N/D | N/D | N/D |
| D6RAR4 | HGFAC | Hepatocyte growth factor activator | 71.5 | 6.7 | N/D | N/D | N/D | N/D | N/D | N/D | N/D | N/D | 15.7 | N/D | N/D | N/D | N/D |
| Q9P225 | DNAH2 | Dynein heavy chain 2, axonemal | 507.7 | 6.0 | N/D | N/D | N/D | N/D | N/D | N/D | N/D | N/D | 18.6 | N/D | N/D | N/D | N/D |
| Q9UBK5 | HCST | Hematopoietic cell signal transducer | 9.5 | 8.6 | N/D | N/D | N/D | N/D | N/D | 24.8 | N/D | N/D | N/D | N/D | N/D | N/D | N/D |
| Q8ND07 | C14orf45 | Uncharacterized protein C14orf45 | 62.0 | 9.1 | N/D | N/D | N/D | N/D | N/D | 21.3 | N/D | N/D | N/D | N/D | N/D | N/D | N/D |
| P02766 | TTR | Transthyretin | 15.9 | 5.5 | N/D | N/D | N/D | N/D | N/D | N/D | N/D | N/D | 15.0 | N/D | N/D | N/D | N/D |
| M0R1K5 | NSUN4 | 5-methylcytosine rRNA methyltransferase NSUN4 | 17.1 | 9.4 | N/D | N/D | N/D | N/D | N/D | N/D | N/D | N/D | 19.5 | N/D | N/D | N/D | N/D |
| Q6EMK4 | VASN | Vasorin | 71.7 | 7.2 | N/D | N/D | N/D | N/D | N/D | N/D | N/D | N/D | 15.6 | N/D | N/D | N/D | N/D |
| Q8TDD2 | SP7 | Transcription factor Sp7 | 45.0 | 8.7 | N/D | N/D | N/D | N/D | N/D | N/D | 16.9 | N/D | N/D | N/D | N/D | N/D | N/D |
| P54132 | BLM | Bloom syndrome protein | 159.0 | 7.3 | N/D | N/D | N/D | N/D | N/D | 21.0 | N/D | N/D | N/D | N/D | N/D | N/D | N/D |
| O75037 | KIF21B | Kinesin-like protein KIF21B | 182.7 | 6.7 | N/D | N/D | N/D | N/D | N/D | N/D | N/D | N/D | 19.0 | N/D | N/D | N/D | N/D |
| Q9H706 | GAREM | GRB2-associated and regulator of MAPK protein | 97.2 | 6.3 | N/D | N/D | N/D | N/D | N/D | N/D | N/D | 23.3 | N/D | N/D | N/D | N/D | N/D |
| Q5RHP9 | C1orf173 | Uncharacterized protein C1orf173 | 168.5 | 4.8 | N/D | N/D | N/D | N/D | N/D | N/D | 17.2 | N/D | N/D | N/D | N/D | N/D | N/D |
| Q8N7X0 | ADGB | Androglobin | 189.7 | 8.5 | N/D | N/D | N/D | N/D | N/D | N/D | N/D | 21.0 | N/D | N/D | N/D | N/D | N/D |
| Q9H0J4 | QRICH2 | Glutamine-rich protein 2 | 180.8 | 6.3 | N/D | N/D | N/D | N/D | N/D | N/D | 14.9 | N/D | N/D | N/D | 17.2 | N/D | 15.0 |
| O00300 | TNFRSF11B | Tumor necrosis factor receptor superfamily member 11B | 46.0 | 8.7 | N/D | N/D | N/D | N/D | N/D | 23.4 | N/D | N/D | N/D | N/D | N/D | N/D | N/D |
| H9KV90 | SHANK1 | SH3 and multiple ankyrin repeat domains protein 1 | 225.9 | 8.5 | N/D | N/D | N/D | N/D | N/D | N/D | N/D | 22.7 | N/D | N/D | N/D | N/D | N/D |
| Q16533 | SNAPC1 | snRNA-activating protein complex subunit 1 | 43.0 | 9.5 | N/D | N/D | N/D | N/D | N/D | N/D | N/D | 19.4 | N/D | N/D | N/D | N/D | N/D |
| Q14671 | PUM1 | Pumilio homolog 1 | 126.5 | 6.4 | N/D | N/D | N/D | N/D | N/D | N/D | 16.4 | N/D | N/D | N/D | N/D | N/D | N/D |
| Q9UIF8 | BAZ2B | Bromodomain adjacent to zinc finger domain protein 2B | 240.5 | 6.1 | N/D | N/D | N/D | N/D | N/D | N/D | 17.3 | N/D | N/D | N/D | N/D | N/D | N/D |
| P02549 | SPTA1 | Spectrin alpha chain, erythrocyte | 280.0 | 5.0 | N/D | N/D | N/D | N/D | N/D | 14.4 | N/D | N/D | N/D | N/D | N/D | N/D | N/D |
| P48634 | PRRC2A | Protein PRRC2A | 228.9 | 9.5 | N/D | N/D | N/D | N/D | N/D | N/D | N/D | N/D | 20.6 | N/D | N/D | N/D | N/D |
| Q9NZI6 | TFCP2L1 | Transcription factor CP2-like protein 1 | 54.6 | 6.5 | N/D | N/D | N/D | N/D | N/D | 21.1 | N/D | N/D | N/D | N/D | N/D | N/D | N/D |
| Q5T0Z8 | C6orf132 | Uncharacterized protein C6orf132 | 124.0 | 9.5 | N/D | N/D | N/D | N/D | N/D | N/D | N/D | N/D | 17.5 | N/D | N/D | N/D | N/D |
| P07738 | BPGM | Bisphosphoglycerate mutase | 30.0 | 6.1 | N/D | N/D | N/D | N/D | N/D | 14.7 | N/D | N/D | N/D | N/D | N/D | N/D | N/D |
| H3BS03 | FANCA | Fanconi anemia group A protein (Fragment) | 17.3 | 8.4 | N/D | N/D | N/D | N/D | N/D | N/D | N/D | 25.1 | N/D | N/D | N/D | N/D | N/D |
| O60760 | HPGDS | Hematopoietic prostaglandin D synthase | 23.3 | 5.5 | N/D | N/D | N/D | N/D | N/D | 20.3 | N/D | N/D | N/D | N/D | N/D | N/D | N/D |
| Q9ULJ7 | ANKRD50 | Ankyrin repeat domain-containing protein 50 | 155.9 | 6.1 | N/D | N/D | N/D | N/D | N/D | 22.1 | N/D | N/D | N/D | N/D | N/D | N/D | N/D |
| P21817 | RYR1 | Ryanodine receptor 1 | 565.2 | 5.2 | N/D | N/D | N/D | N/D | N/D | N/D | N/D | 19.6 | N/D | N/D | N/D | N/D | N/D |
| Q4KMZ1-3 | IQCC | Isoform 3 of IQ domain-containing protein C | 62.0 | 8.7 | N/D | N/D | N/D | N/D | N/D | N/D | N/D | N/D | 16.7 | N/D | N/D | N/D | N/D |
| Q9BZ29 | DOCK9 | Dedicator of cytokinesis protein 9 | 236.4 | 7.3 | N/D | N/D | N/D | N/D | N/D | N/D | 18.2 | N/D | N/D | N/D | 18.4 | N/D | 19.0 |
| Q12788 | TBL3 | Transducin beta-like protein 3 | 89.0 | 6.4 | N/D | N/D | N/D | N/D | N/D | N/D | N/D | N/D | 16.3 | N/D | N/D | N/D | N/D |
| Q6PGQ7 | BORA | Protein aurora borealis | 61.2 | 4.8 | N/D | N/D | N/D | N/D | N/D | 24.1 | N/D | N/D | N/D | N/D | N/D | N/D | N/D |
| P59923 | ZNF445 | Zinc finger protein 445 | 119.0 | 9.5 | N/D | N/D | N/D | N/D | N/D | N/D | N/D | 23.6 | N/D | N/D | N/D | N/D | N/D |
| P24855 | DNASE1 | Deoxyribonuclease-1 | 31.4 | 4.7 | N/D | N/D | N/D | N/D | N/D | N/D | 16.0 | N/D | N/D | N/D | N/D | N/D | N/D |
| B4DH94 | NAV3 | Neuron navigator 3 | 24.8 | 5.2 | N/D | N/D | N/D | N/D | N/D | N/D | N/D | N/D | 18.0 | N/D | N/D | N/D | N/D |
| O60287 | URB1 | Nucleolar pre-ribosomal-associated protein 1 | 254.4 | 6.0 | N/D | N/D | N/D | N/D | N/D | N/D | N/D | N/D | 17.3 | N/D | N/D | N/D | N/D |
| Q9H7Z3 | C14orf102 | UPF0614 protein C14orf102 | 132.7 | 7.7 | N/D | N/D | N/D | N/D | N/D | 21.6 | N/D | N/D | N/D | N/D | N/D | N/D | N/D |
| P06727 | APOA4 | Apolipoprotein A-IV | 45.4 | 5.3 | N/D | N/D | N/D | N/D | N/D | N/D | N/D | N/D | N/D | 20.5 | N/D | N/D | N/D |
| P15924 | DSP | Desmoplakin | 331.8 | 6.4 | N/D | N/D | N/D | N/D | N/D | N/D | N/D | N/D | N/D | N/D | N/D | 17.7 | N/D |
| P05155 | SERPING1 | Plasma protease C1 inhibitor | 55.2 | 6.1 | N/D | N/D | N/D | N/D | N/D | N/D | N/D | N/D | N/D | 20.0 | N/D | N/D | N/D |
| Q5D862 | FLG2 | Filaggrin-2 | 248.1 | 8.5 | N/D | N/D | N/D | N/D | N/D | N/D | N/D | N/D | N/D | N/D | N/D | 16.7 | N/D |
| P04406 | GAPDH | Glyceraldehyde-3-phosphate dehydrogenase | 36.1 | 8.6 | N/D | N/D | N/D | N/D | N/D | N/D | N/D | N/D | N/D | N/D | N/D | 18.0 | N/D |
| P08603 | CFH | Complement factor H | 139.1 | 6.2 | N/D | N/D | N/D | N/D | N/D | N/D | N/D | N/D | N/D | 19.8 | N/D | N/D | N/D |
| P10909 | CLU | Clusterin | 52.5 | 5.9 | N/D | N/D | N/D | N/D | N/D | N/D | N/D | N/D | N/D | 20.0 | N/D | N/D | N/D |
| Q15828 | CST6 | Cystatin-M | 16.5 | 8.3 | N/D | N/D | N/D | N/D | N/D | N/D | N/D | N/D | N/D | N/D | N/D | N/D | 18.2 |
| Q8NF91 | SYNE1 | Nesprin-1 | 1011.0 | 5.4 | N/D | N/D | N/D | N/D | N/D | N/D | N/D | N/D | N/D | N/D | 16.1 | N/D | 17.2 |
| Q8IXH8 | CDH26 | Cadherin-like protein 26 | 95.3 | 5.6 | N/D | N/D | N/D | N/D | N/D | N/D | N/D | N/D | N/D | 18.8 | 19.1 | 20.2 | 19.6 |
| P08294 | SOD3 | Extracellular superoxide dismutase [Cu-Zn] | 25.9 | 6.1 | N/D | N/D | N/D | N/D | N/D | N/D | N/D | N/D | N/D | N/D | 16.8 | N/D | N/D |
| Q02413 | DSG1 | Desmoglein-1 | 113.7 | 4.9 | N/D | N/D | N/D | N/D | N/D | N/D | N/D | N/D | N/D | N/D | N/D | 18.2 | N/D |
| Q03591 | CFHR1 | Complement factor H-related protein 1 | 37.7 | 7.4 | N/D | N/D | N/D | N/D | N/D | N/D | N/D | N/D | N/D | 18.0 | N/D | N/D | N/D |
| P31944 | CASP14 | Caspase-14 | 27.7 | 5.4 | N/D | N/D | N/D | N/D | N/D | N/D | N/D | N/D | N/D | N/D | N/D | 17.2 | N/D |
| P07998 | RNASE1 | Ribonuclease pancreatic | 17.6 | 9.1 | N/D | N/D | N/D | N/D | N/D | N/D | N/D | N/D | N/D | N/D | 16.6 | 16.5 | 18.0 |
| O00468 | AGRN | Agrin | 214.8 | 6.0 | N/D | N/D | N/D | N/D | N/D | N/D | N/D | N/D | N/D | N/D | N/D | N/D | 16.0 |
| Q99758 | ABCA3 | ATP-binding cassette sub-family A member 3 | 191.4 | 7.6 | N/D | N/D | N/D | N/D | N/D | N/D | N/D | N/D | N/D | 20.5 | N/D | N/D | N/D |
| Q6ZVS7 | FAM183B | Protein FAM183B | 16.2 | 8.1 | N/D | N/D | N/D | N/D | N/D | N/D | N/D | N/D | N/D | 19.1 | N/D | N/D | N/D |
| P02741 | CRP | C-reactive protein | 25.0 | 5.5 | N/D | N/D | N/D | N/D | N/D | N/D | N/D | N/D | N/D | 18.8 | N/D | N/D | N/D |
| Q5W0A0 | FAM194B | Protein FAM194B | 81.7 | 4.7 | N/D | N/D | N/D | N/D | N/D | N/D | N/D | N/D | N/D | N/D | N/D | 21.7 | N/D |
| Q12805 | EFEMP1 | EGF-containing fibulin-like extracellular matrix protein 1 | 54.6 | 5.0 | N/D | N/D | N/D | N/D | N/D | N/D | N/D | N/D | N/D | 17.8 | N/D | N/D | N/D |
| Q5T749 | KPRP | Keratinocyte proline-rich protein | 64.1 | 8.7 | N/D | N/D | N/D | N/D | N/D | N/D | N/D | N/D | N/D | N/D | N/D | 18.1 | N/D |
| P59510 | ADAMTS20 | A disintegrin and metalloproteinase with thrombospondin motifs 20 | 214.7 | 7.0 | N/D | N/D | N/D | N/D | N/D | N/D | N/D | N/D | N/D | N/D | N/D | 18.7 | N/D |
| Q15643 | TRIP11 | Thyroid receptor-interacting protein 11 | 227.6 | 5.2 | N/D | N/D | N/D | N/D | N/D | N/D | N/D | N/D | N/D | N/D | N/D | N/D | 16.2 |
| Q9Y276 | BCS1L | Mitochondrial chaperone BCS1 | 47.5 | 8.6 | N/D | N/D | N/D | N/D | N/D | N/D | N/D | N/D | N/D | N/D | N/D | 16.1 | N/D |
| Q02218 | OGDH | 2-oxoglutarate dehydrogenase, mitochondrial | 115.9 | 6.4 | N/D | N/D | N/D | N/D | N/D | N/D | N/D | N/D | N/D | N/D | N/D | 19.6 | N/D |
| Q92545 | TMEM131 | Transmembrane protein 131 | 205.1 | 8.7 | N/D | N/D | N/D | N/D | N/D | N/D | N/D | N/D | N/D | N/D | N/D | 17.0 | N/D |
| O60285 | NUAK1 | NUAK family SNF1-like kinase 1 | 74.3 | 9.0 | N/D | N/D | N/D | N/D | N/D | N/D | N/D | N/D | N/D | N/D | N/D | N/D | 15.2 |
| O00567 | NOP56 | Nucleolar protein 56 | 66.1 | 9.2 | N/D | N/D | N/D | N/D | N/D | N/D | N/D | N/D | N/D | N/D | 16.8 | N/D | N/D |
| Q5HYC2 | KIAA2026 | Uncharacterized protein KIAA2026 | 228.1 | 9.2 | N/D | N/D | N/D | N/D | N/D | N/D | N/D | N/D | N/D | 17.7 | N/D | N/D | N/D |
| P37173 | TGFBR2 | TGF-beta receptor type-2 | 64.6 | 5.6 | N/D | N/D | N/D | N/D | N/D | N/D | N/D | N/D | N/D | N/D | N/D | 17.6 | N/D |
| P07358 | C8B | Complement component C8 beta chain | 67.0 | 8.5 | N/D | N/D | N/D | N/D | N/D | N/D | N/D | N/D | N/D | 19.0 | N/D | N/D | N/D |
| A8MRT5 | 5 SV | Putative NPIP-like protein LOC100132247 | 126.5 | 10.1 | N/D | N/D | N/D | N/D | N/D | N/D | N/D | N/D | N/D | N/D | 15.3 | N/D | N/D |
| Q12967 | RALGDS | Ral guanine nucleotide dissociation stimulator | 100.6 | 5.5 | N/D | N/D | N/D | N/D | N/D | N/D | N/D | N/D | N/D | N/D | 16.8 | N/D | N/D |
| O76039 | CDKL5 | Cyclin-dependent kinase-like 5 | 115.5 | 9.6 | N/D | N/D | N/D | N/D | N/D | N/D | N/D | N/D | N/D | 23.3 | N/D | N/D | N/D |
| Q12830 | BPTF | Nucleosome-remodeling factor subunit BPTF | 338.3 | 6.2 | N/D | N/D | N/D | N/D | N/D | N/D | N/D | N/D | N/D | N/D | N/D | N/D | 16.9 |
| Q9UPN7 | PPP6R1 | Serine/threonine-protein phosphatase 6 regulatory subunit 1 | 96.7 | 4.5 | N/D | N/D | N/D | N/D | N/D | N/D | N/D | N/D | N/D | N/D | N/D | 14.9 | N/D |
| Q7Z2Y5 | NRK | Nik-related protein kinase | 178.5 | 5.9 | N/D | N/D | N/D | N/D | N/D | N/D | N/D | N/D | N/D | 23.2 | N/D | N/D | N/D |
| P11182 | DBT | Lipoamide acyltransferase component of branched-chain alpha-keto acid dehydrogenase complex,  mitochondrial | 53.5 | 8.7 | N/D | N/D | N/D | N/D | N/D | N/D | N/D | N/D | N/D | N/D | N/D | 18.5 | N/D |
| Q8TF46 | DIS3L | DIS3-like exonuclease 1 | 120.8 | 6.1 | N/D | N/D | N/D | N/D | N/D | N/D | N/D | N/D | N/D | N/D | 21.0 | 22.9 | 20.7 |

N/D: non detected
