# Supplementary material for: Potential urine proteomics biomarkers for primary nephrotic syndrome
Source: Clin Proteomics. 2017 May 16;14:18. doi: 10.1186/s12014-017-9153-1 (PMC5434615; doi:10.1186/s12014-017-9153-1)
Supplement: Supplementary file 3 — Additional file 3: Table S2. Differently expressed 22 proteins in MCD, FSGS and MN. [file 12014_2017_9153_MOESM3_ESM.docx]

Additional file 3: Table 2. List of 22 candidate biomarkers in nephrotic syndrome

| Accession number | Gene symbol | MW | *pI* | MCD | MN | FSGS |
| --- | --- | --- | --- | --- | --- | --- |
| P02768 | ALB | 69.4 | 5.9 | 19.7 ± 3.5^*^ | N/D | 18.1 ± 3.0 |
| P02774 | GC | 53.0 | 5.4 | 21.6 ± 2.2^*^ | N/D | 19.7 ± 2.8^‡^ |
| P01009 | SERPINA1 | 46.7 | 5.4 | 19.8 ± 2.1^*^ | N/D | 19.7 ± 4.1 |
| P43652 | AFM | 69.1 | 5.6 | 20.8 ± 0.8^*^ | 19.4 ± 1.0^†^ | 18.9 ± 0.8 |
| P02790 | HPX | 51.7 | 6.6 | 20.1 ± 2.8 | 17.3 ± 1.4 | 20.2 ± 2.2^‡^ |
| P02760 | AMBP | 39.0 | 6.0 | 20.6 ± 1.0 | 18.9 ± 2.0 | 21.4 ± 0.4^‡^ |
| Q96PD5 | PGLYRP2 | 62.2 | 7.3 | 19.7 ± 1.9^*^ | 19.4^a^ | 18.3 ± 1.4 |
| P02749 | APOH | 38.3 | 8.3 | 21.6^a^ | 14.5 ± 1.5 | 17.7 ± 2.4^‡^ |
| P08571 | CD14 | 40.1 | 5.8 | 18.6 ± 1.8^*^ | 17.0^a^ | 17.8 ± 1.3 |
| P08185 | SERPINA6 | 45.1 | 5.6 | 19.4 ± 1.5 | 19.0 ± 2.0^†^ | 16.9 ± 0.5 |
| P04264 | KRT1 | 66.0 | 8.2 | 18.9 ± 3.0 | 17.1 ± 0.2 | 19.0 ± 1.7^‡^ |
| P02748 | C9 | 63.2 | 5.4 | 19.7 ± 2.0^*^ | N/D | 18.7 ± 3.4 |
| P43251 | BTD | 61.1 | 5.8 | 19.4 ± 1.1 | 18.5 ± 2.4^†^ | 17.4^a^ |
| P07911 | UMOD | 69.8 | 5.1 | N/D | 18.1 ± 1.8^*^ | 17.0 ± 0.1 |
| P05543 | SERPINA7 | 46.3 | 5.9 | N/D | 19.2 ± 1.9^*‡^ | N/D |
| P13987 | CD59 | 14.2 | 6.0 | N/D | 19.7 ± 0.2^†^ | 19.4 ± 0.8 |
| P16070 | CD44 | 81.5 | 5.1 | N/D | 17.0 ± 1.7^*‡^ | N/D |
| P10809 | HSPD1 | 61.1 | 5.7 | N/D | 16.8 ± 0.1^†^ | 17.1 ± 1.4 |
| P05451 | REG1A | 18.7 | 5.7 | N/D | 17.8^a^ | 17.4 ± 1.7^†^ |
| Q8IXH8 | CDH26 | 95.3 | 5.6 | N/D | N/D | 19.4 ± 0.6^†‡^ |
| P07998 | RNASE1 | 17.6 | 9.1 | N/D | N/D | 17.0 ± 0.9^†‡^ |
| Q8TF46 | DIS3L | 120.8 | 6.1 | N/D | N/D | 21.5 ± 1.2^†‡^ |

The results are transformed to log2 based-scale for peak intensity of protein.

N/D (none detected), a: detected value in one sample

*p<0.05 MCD vs. MN, †p<0.05 MCD vs. FSGS, ‡p<0.05 MN vs. FSGS
